# High content screening application for cell-type specific behaviour in heterogeneous primary breast epithelial subpopulations

**DOI:** 10.1186/s13058-016-0681-9

**Published:** 2016-02-09

**Authors:** Rebecca L. Johnston, Leesa Wockner, Amy E. McCart Reed, Adrian Wiegmans, Georgia Chenevix-Trench, Kum Kum Khanna, Sunil R. Lakhani, Chanel E. Smart

**Affiliations:** 1 The University of Queensland, UQ Centre for Clinical Research, Brisbane, Queensland 4029 Australia; 2 QIMR Berghofer Medical Research Institute, Brisbane, Queensland 4029 Australia; 3 Pathology Queensland, Royal Brisbane and Women’s Hospital, Brisbane, Queensland 4029 Australia; 4 The University of Queensland, School of Medicine, Brisbane, Queensland 4029 Australia

**Keywords:** High-content immunofluorescence, Breast, Mixed cell cultures, DNA damage, Ionising radiation

## Abstract

**Background:**

The complex interaction between multiple cell types and the microenvironment underlies the diverse pathways to carcinogenesis and necessitates sophisticated approaches to *in vitro* hypotheses testing. The combination of mixed culture format with high content immunofluorescence screening technology provides a powerful platform for observation of cell type specific behavior.

**Methods:**

We have developed a versatile, high-throughput method for assessing cell-type specific responses. In addition to the specificity and sensitivity offered traditionally by immunofluorescent detection in flow cytometry, the ‘in-cell’ analysis method we describe provides the added benefits of higher throughput and the ability to analyse protein subcellular localisation *in situ*. Furthermore, elimination of the cell dissociation step allows for more immediate analysis of responses to specific extrinsic stimuli. We applied this method to investigate ionising radiation treatment response in normal breast epithelial cells, measuring growth rate, cell cycle response and double-strand DNA breaks.

**Results:**

The ‘in-cell’ analysis approach elucidated several interesting donor and cell-type specific differences. Notably, in response to ionizing radiation we observed differential expression in luminal and basal-like cells of a member of the APOBEC enzyme family, recently identified as a critical driver of an oncogenic signature. Our findings suggest that this enzyme is active in the normal breast epithelium during DNA damage response.

**Conclusions:**

We demonstrate the practical application of a new method for assessing cell-type specific change in mixed cultures, especially the analysis of normal primary cultures, overcoming a major technical issue of dissecting the response of multiple cell types in a heterogeneous population.

**Electronic supplementary material:**

The online version of this article (doi:10.1186/s13058-016-0681-9) contains supplementary material, which is available to authorized users.

## Background

It is plausible that some aspects of the breast cancer heterogeneity may be explained by their histogenesis from different normal subpopulations. Growing interest in the cellular origins of different breast cancer subtypes has prompted investigations into the different subpopulations of the normal breast epithelia and their differentiation hierarchy [1–5]. Several groups have, for instance, demonstrated a probable luminal progenitor cell origin for basal-like breast cancer [4, 6, 7]; however, this is still debated, and the cellular origin for other breast cancer subtypes remain to be determined. The specific factors that initiate cancer progression in the normal cell, and how they might be different between normal cell subtypes in the one tissue, are also still poorly understood. This is partly due to a paucity of models (particularly human) that permit investigation of cell-type specific hypotheses. Emerging functional studies of subpopulation-specific mammary epithelial biology indicate that the luminal progenitor compartment exhibits telomere dysfunction which might be a link to an unstable genome and tumourigenesis occurring in this cell type [8], which represents a great step forward in our understanding of the early steps of carcinogenesis in the breast.

The molecular and cellular mechanisms underlying why one cell type might be more susceptible to transformation are yet to be elucidated. There is general agreement that the vast majority of breast cancers are derived from the luminal subpopulations, whilst the basal cells may contribute only to more rare subtypes such as myoepithelioma and metaplastic carcinoma [2]. Comparative studies of normal tissue adjacent to tumours show that neighbouring normal luminal and basal cells share the same initial genetic changes as the tumour [9]. This could imply that basal cells somehow ‘resist’ transformation, whilst luminal cells can accumulate further change and ultimately form a tumour [10]. This is interesting given that the different cell types are genetically identical and reside together in the same tissue, yet their inherently different biology and microenvironment might contribute to differences in transformation susceptibility. Thus we can hypothesise that the interplay between microenvironment and cell type drives oncogenic change at different rates within a heterogeneous population.

**Fig. 1:**
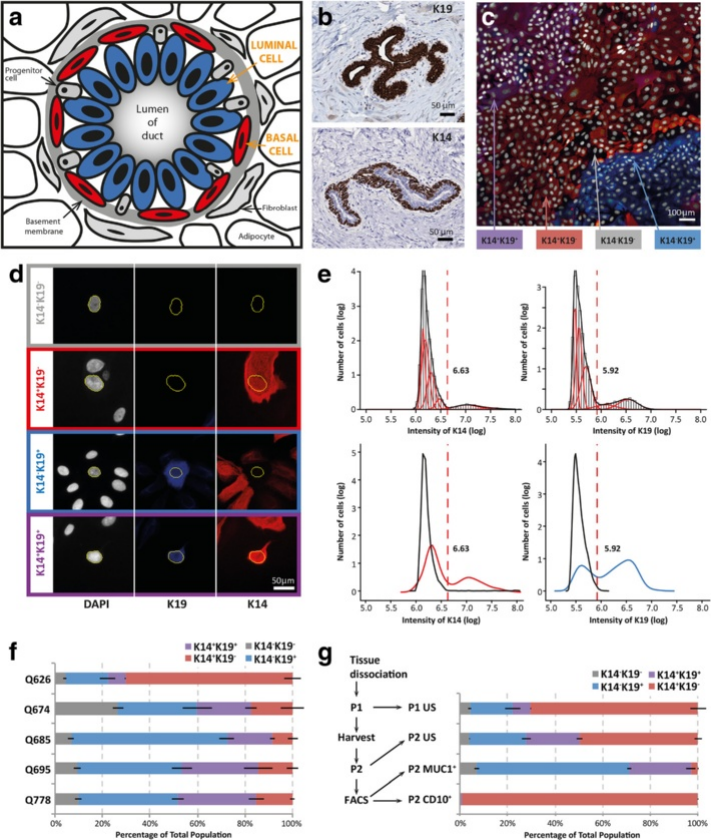
High-content screening of normal human mammary epithelial cells (hMECs). **a** Schematic representation of a normal human mammary duct in vivo showing the major cell types: basal cells (*red*) and luminal cells (*blue*). **b** Light micrographs of immunohistochemical staining of K19 and K14 on FFPE sections of normal human breast delineating luminal and basal cells respectively. **c** Representative image of immunofluorescence staining of primary hMECs acquired using the IN Cell Analyser 2000 (GE Healthcare, Silverwater, NSW, Australia) at 20× magnification. Cells were stained for K14 (*red*) and K19 (*blue*) and counterstained with DAPI (*white*). Areas of the four possible cell phenotypes are indicated (*arrows*): double negative or K14^−^/K19^−^; double positive or K14^+^/K19^+^; K14^+^ (K14^+^/K19^−^); and K19^+^ (K14^−^/K19^+^). **d** Identification of cell phenotype on a single cell basis using digital image analysis. The image analysis protocol defines cells based on nuclear segmentation (*first column*), then subsequently measures the average intensity of K14 (*second column*) and K19 (*third column*) within each cell. The four possible outcomes for K14 and K19 are shown by row. **e** Intensity distributions for K14 (*left panels*) and K19 (*right p*anels) in primary hMEC culture of negative secondary only control (*black*) and stained samples plotting their respective cytokeratin distribution (K14, *red*; K19, *blue*). **f** Subpopulation frequency of the four cell phenotypes in the first passage of hMECs for five donors. Mean subpopulation frequencies across (*n* = 5–6) technical replicates were calculated as the average percentage of total cell number within each well for each of the five individual donors (± standard error of the mean (SEM)). **g** Subpopulation frequency with passage and enrichment using FACS (Fluorescence-activated cell sorting). Cells from reduction mammoplasties were dissociated and cultured in vitro (P1). After 5–7 days, cells were harvested and sorted based on expression of CD10 and MUC1 using FACS. CD10^+^, MUC1^+^ and unsorted (*US*) cells were cultured for 5 days (P2) before fixation and analysis of population heterogeneity. Mean subpopulation frequencies were calculated as the average percentage of total cell number within each well for each sample (± SEM). *DAPI* 4′,6-diamidino-2-phenylindole, *FACS* fluorescence-activated cell sorting, *K14* cytokeratin 14, *K19* cytokeratin 19, *P1* passage 1, *P2* passage 2

To address this hypothesis, the biological advantages of working with primary human breast tissue as opposed to mouse models or human breast cancer-derived cell lines are numerous. Fresh dissociations and primary cultures of normal breast epithelial tissue retain a large degree of the desirable cellular heterogeneity of the in vivo gland. Cytokeratins 19 and 14 (K19 and K14), which delineate the major divisions of the luminal and basal epithelium in vivo (Fig. 1a, b), are retained in mixed primary cultures (Fig. 1c). Because mixed cell phenotypes in primary culture can, however, ‘confound’ or dilute any overall observations if the culture is treated as a pool, a reliable method of distinguishing cell types is needed. Whilst this is possible with flow cytometry, the method necessitates the disruption of the cell from the microenvironment and neighbouring cells that the mixed primary culture has allowed. A more versatile method which allows ‘in cell’ analysis of mixed cultures would give the field much greater flexibility to analyse cell-type specific effects with greater accuracy (not requiring harvesting and fixation for cytometry). This method would not only allow the analysis of response to different stimuli and microenvironmental changes, but would also allow cell-type specific analysis of gene function. We have developed a high-throughput, high-content method for screening cell-type specific effects in mixed primary breast epithelial culture and applied it to the specific question of response to ionising radiation (IR), a known environmental risk factor and therapy for breast cancer.

## Materials and methods

### Reduction mammoplasty samples

Fresh reduction mammoplasty samples from 12 donors with appropriate written consent and approval by local human research ethics committees. Glandular tissue was cut into small pieces (approximately 1 cm^3^) and prepared and cultured as described previously by Smart et al. [11]. Samples were digested overnight with agitation at 37 °C and 5 % CO_2_ in digestion media containing Dulbecco’s modified Eagle’s medium:F12 (1:1; Gibco) supplemented with 5 % foetal bovine serum (FBS; Gibco, Life Technologies Australia Pty Ltd., Scoresby, Australia), 1× antibiotic/antimycotic (Gibco), 2.5 μg/ml fungizone (Gibco), 200 U/ml collagenase type I-A (Sigma, Castle Hill, NSW, Australia) and 100 U/ml hyaluronidase I-S (Sigma). Organoids were obtained by centrifugal separation [12] (1 minute at 80 × *g*). To generate single cells, organoids were dissociated using TrypLE Express (Gibco) for 5–10 minutes, quenched in Hank’s Balanced Salt Solution (HBSS; Gibco) supplemented with 2 % FBS and subsequently filtered through a 40 μm cell strainer (BD Falcon, North Ryde, NSW, Australia).

### Primary cell culture and treatment with IR

Single mammary epithelial cells were cultured in black 96-well optical-bottom plates (Nunc, Life Technologies Australia Pty Ltd., Scoresby, VIC,Australia) in adherent conditions at 37 °C and 5 % CO_2_ using E93 media [13], containing Ham’s F12 nutrient mixture (Gibco), 5 % FBS, 1× antibiotic/antimycotic, 10 ng/ml epidermal growth factor (Sigma), 5 mg/ml insulin (Sigma), 1 μg/ml hydrocortisone (Sigma) and 100 ng/ml cholera toxin (Sigma). Cells were seeded at 2000, 4000 and 8000 cells/well with three to six (optimally five) technical replicates per cell dilution. All cell counts were performed in triplicate using a Countess Automated Cell Counter (Invitrogen, Life Technologies Australia Pty Ltd., Scoresby, VIC, Australia) prior to seeding. Cells were treated with IR using the MDS Nordian (Macquarie Park NSW, AUSTRALIA) Gammacell 40 Exactor. To fix, cells were rinsed briefly in cytoskeleton buffer (10 mM Hepes adjusted to pH 7.4 with KOH, 300 mM sucrose, 100 mM NaCl, 3 mM MgCl_2_), fixed in 100 % ice-cold methanol for 5 minutes and washed three times in 1× PBS. Seeding densities showing 30–50 % confluency – that is, in the log phase of growth at the time of IR treatment (5 days) – were chosen for the experiments.

For the analysis of first-passage cultures, including baseline assessment of cytokeratin heterogeneity and DNA damage response, cells were treated with 0, 1, 2 and 5 Gy IR after 5 or 6 days of growth, and were fixed at 0, 1, 2, 6, 24 and 48 hours post IR.

For subpopulation enrichment experiments coupled with fluorescence-activated cell sorting (FACS) (see later), passage 1 (P1) cells were fixed at 7 days of growth (same day as FACS experiment). Passage 2 (P2, post-sort) cell subpopulations, including CD10^+^, MUC1^+^ and unsorted cells, were cultured immediately post FACS at 4000 cells/well into 96-well plates and fixed at 5 days. In addition to 96-well plates, P1 cells were cultured in 10–15 T75 flasks for FACS at 1.5 × 10^6^ cells/flask, and P2 CD10^+^ and MUC1^+^ cells were each cultured in six T25 flasks at 1 × 10^5^ cells/flask for IR treatment and RNA extraction (see later). After 5 days of culture, P2 cells were irradiated at 0, 2 and 5 Gy and then collected 2 and 24 hours post IR in RLT lysis buffer (AllPrep DNA/RNA Mini Kit; Qiagen) as per the manufacturer’s protocol (12 samples per donor; see Fig. 6a).

### Cell staining and flow cytometry

Immunohistochemical staining of normal human breast tissue was performed as described previously [11]. For subpopulation enrichment experiments, after 7 days in primary culture P1 normal breast epithelial cells from the T75 flasks were washed twice in phosphate-buffered saline (PBS), treated with Versene (Gibco) for 10 minutes and then treated with TrypLE Express for 5–10 minutes. Suspended cells were collected and quenched in HBSS supplemented with 2 % FBS. Cells were labelled with the BD Bioscience antibodies CD10-phycoerythrin (PE)-Cy5 (1:80 dilution), MUC1-fluorescein isothiocyanate (FITC) (1:100 dilution), CD31-PE (1:100 dilution), CD45-PE (1:100 dilution), CD140b-PE (1:100 dilution), and Sytox Blue (1:1000 dilution; Molecular Probes) at a concentration of 2 × 10^6^ cells/ml for 15 minutes on ice. Cells were sorted using a BD FACS Aria II Cell Sorter with the strategy depicted in Fig. 1g. Mammary epithelial cell subpopulations of CD10^+^-sorted, MUC1^+^-sorted and ‘unsorted’ cells (live cells processed through the Aria) were obtained and immediately cultured as already specified.

### Immunofluorescence

Fixed cells were blocked using FBT blocking buffer (5 % FBS, 1 % bovine serum albumin, 0.05 % Tween-20, 10 mM Tris pH 7.5, 100 mM MgCl_2_) for 30 minutes prior to addition of primary antibodies. Cells were stained with the following primary antibodies diluted in FBT for 60 minutes: polyclonal rabbit anti-K5 AF138 (1:100; Covance, Macquarie Park NSW Australia), IgG3 mouse anti-K14 LL002 (1:50; Novocastra, Leica Biosystems, North Ryde, NSW, Australia), IgG1 mouse anti-K8/18 5D3 (1:100; Novocastra), IgG2a mouse anti-K19 A53-B/A2 (1:50; AbD Serotec, Biorad, Gladesville, NSW, Australia) and IgG1 mouse-anti γH2AX (1:300; BD Biosciences, Life Technologies Australia Pty Ltd., Scoresby, VIC, Australia). Following primary antibody incubation, cells were washed three times in 1× PBS and then incubated with the following secondary antibodies diluted in FBT for 30 minutes: Alexa fluor anti-mouse IgG1 488 (1:400), anti-mouse IgG3 594 (1:400), anti-mouse IgG2a 633 (1:200) and nuclear counterstain 4′,6-diamidino-2-phenylindole (DAPI, 0.1 μg/ml; Molecular Probes). Secondary only control wells (including DAPI) were included for every time point and/or sorted subpopulation. Stained wells were mounted in 75 % glycerol in PBS. For EdU experiments, we used the Click-iT EdU Alexa Fluor 488 HCS Assay (Molecular Probes) prior to primary antibody addition. Cells were treated with 10 μM of EdU, fixed 4 hours post-EdU treatment and then EdU was detected according to the manufacturer’s protocol.

### Image acquisition

Immunofluorescent images were acquired using the IN Cell Analyser 2000 (INCA; GE Healthcare, Silverwater, NSW, Australia). Each plate was acquired with the following settings: 20× objective; 0.25 SAC collar; four wavelengths; 2-D imaging mode; 2 × 2 binning; QUAD1 polychroic; flat field correction; 25 fields per well, 5 × 5 fixed layout, 100 μm distance between fields; and hardware autofocus alone. DAPI, FITC, Cy3 and Cy5 excitation and emission filters were used to image DAPI, Alexa fluor 488, 594 and 633, respectively. Focus offset and exposure times were optimised for each donor using the visuals histogram to ensure maximum dynamic range of intensity without overexposing the sample.

### Image analysis

Image analysis was performed using Developer Toolbox v1.9 (GE Healthcare, Silverwater, NSW, Australia). Cell targets were segmented based on DAPI intensity (nuclear segmentation) and nuclear form factor (>0.8, where 1.0 is a perfect circle). Post-processing procedures including watershed clump breaking, sieve (defined range of permitted target areas) and border object removal (removal of targets on edge of acquired fields) were performed to maximise identification of single cells. Cell-by-cell measurements were recorded by applying nuclear segmentation (DAPI channel) to the corresponding FITC, Cy3 and Cy5 channel images from each field, as shown in Fig. 1d. For cell counts and cell cycle analysis, target count, nucleus area (μm^2^), total DAPI intensity (‘mass’) and average DAPI intensity (‘density’) were recorded. For cell phenotype classification, the average intensity of FITC, Cy3 and Cy5 within the area defined by nuclear segmentation were recorded. Data outputs were exported to .xls files for analysis.

### Phenotype classification, cell cycle profile assignment and statistical analyses

All output data from image analyses were imported and analysed using the R environment for statistical computing (www.r-project.org).

#### Phenotype

To determine the threshold (cut-off point) for positivity of each cytokeratin, the natural logarithm of average pixel intensity for each fluorochrome (FITC, Cy3, Cy5) per cell was plotted on a row-by-row basis (representing one technical replicate), together with the cell-by-cell values from the appropriate secondary only control well. A two-component mixture model [14] was applied to define the mixture of the positive and negative population modelled by a mixture of normal distributions (Additional file 1: Figure S1). The secondary only control population was used to identify the null distribution as the combination of components that had mean density less than the first intersection of distributions after the secondary only population approaches zero (Fig. 1e).

#### Cell cycle

The total DAPI intensity per cell per well (technical replicate) was plotted and cell cycle phases were assigned using mixtures of fitted *t* distributions and standard deviations between the two peaks to determine four cut-off points (intersection of sub-G_1_/G_1_, G_1_/S, S/G_2_ and G_2_/post-G_2_).

#### γH2AX

γH2AX was normalised by subtracting the secondary only control well γH2AX expression on a row-by-row basis. To normalise between plates, a linear model (using the rlm function in the MASS package in R) was fitted to the γH2AX expression at time zero. Plate and column effects (and interaction effects if applicable) were then subtracted from all experimental wells.

### Statistical analyses

After application of appropriate thresholds per treatment, sample populations were assigned to phenotype (K19^+^, K14^+^, double negative (DN, K14^−^/K19^−^) and double positive (DP, K14^+^/K19^+^)) and cell cycle (sub-G_1_, G_1_, S, G_2_ and post-G_2_). Average frequencies for each category over technical replicates (in most cases (*n* = 5)) were plotted, with standard error. Mean γH2AX values of technical replicates were also plotted. Statistical comparisons were performed using ordinary one-way analysis of variance (ANOVA) unless otherwise indicated. Proportional Venn diagrams were drawn using eulerAPE online software (http://www.eulerdiagrams.org/eulerAPE/).

Doubling times, and consequently growth rates, were estimated by calculating the log (base 2) transformation of cell counts within each well for each time point (*t* = 0, 1, 2, 6, 24, 48). The following linear equation was then fitted via least-squares regression:

$$ \mathrm{l}\mathrm{o}{\mathrm{g}}_2\left(\mathrm{coun}{\mathrm{t}}_{(t)}\right)=a+bt $$

The doubling time was taken to be 2^*b*^ and the growth curve was then defined as count_(*t*)_ = 2^*a*^ *2* ^*tb*^.

To determine for an individual donor whether the change in the proportion of cells differed over time for each treatment (0 and 5 Gy), an ANOVA method known as a split-plot analysis was performed. Split-plot analysis is used when the treatment and plate are completely confounded because it is impractical to apply multiple treatments to a single plate. The interaction between time and plate tests the hypothesis that the change in the proportion over time cells is equivalent across treatments. Time was treated as a continuous variable and hence the null assumption was that the relative proportion of each phenotype was consistent across time.

### RNA extraction

RNA was extracted from the 12 × P2 subpopulations (CD10^+^ and MUC1^+^ treated with 0, 2, and 5 Gy IR and collected 2 and 24 hours post IR; see Fig. 6a) for five donors using the QIAGEN AllPrep DNA/RNA Mini Kit as per the manufacturer’s protocol. RNA yield was measured using the NanoDrop 2000 (Thermo Scientific, Life Technologies Australia Pty Ltd., Scoresby, VIC, Australia), and RNA integrity was confirmed for microarrays using the Agilent Bioanalyser 2100 (Agilent Technologies, Santa Clara, CA, USA).

### Gene expression profiling

Gene expression microarray experiments were performed using the Illumina HT-12 v4 arrays for a total of five donors. A one-colour approach was used, where each donor set of 12 samples was hybridised onto the same array, and all five arrays were processed simultaneously. A total of 250 ng of RNA was used per sample for cRNA production using the TotalPrep RNA Amplification Kit (Ambion, Life Technologies Australia Pty Ltd., Scoresby, VIC, Australia 10.1186/s13058-016-0681-9). Array hybridisation was performed using the Expression BeadChip Kit (Illumina, Scoresby, VIC, Australia), and 750 ng of biotin-labelled cRNA was hybridised to the HT-12 array. Scanning was performed using an Illumina BeadArray Reader.

### Microarray data analysis

Array data were processed using the R package ‘limma’ [15]. Data from all five donors were background corrected using the normal and exponential convolution model method (normexp), quantile normalised and log_2_ transformed. Data are available through GEO [GEO:GSE67595]. Only probes with an Illumina Detection Score >0.95 in >50 % of the samples were kept for further analyses. This data filtering method kept 19,472 probes (41 % of total). A hypothesis test analogous to the classical paired *t* tests [16] was performed across the samples to find differentially expressed genes, defined by a log fold-change ≥0.5 or ≤ −0.5 and a B score >0 (see Fig. 6a). Unsupervised principal components analysis (PCA) was applied to gene expression profiles from all 60 samples across the five donors. The R package ‘rgl’ was used to plot the PCA results by projecting each sample to the first three principal components. To identify overrepresented biological pathways in respective samples, the full filtered gene lists of 19,472 probes with fold-change in MUC1-sorted and CD10-sorted cultures were uploaded into and analysed in ingenuity pathway analysis.

### Quantitative reverse transcriptase PCR

RNA was isolated from sorted cultured cells using a RNA isolation kit (Qiagen, Chadstone Centre, VIC, Australia). SuperScript III Reverse Transcriptase (Invitrogen, Mulgrave, VIC, Australia) was used to generate cDNA from 2 μg of RNA. The samples were then incubated at 42 °C for 50 minutes and the reverse transcriptase (RT) enzyme was inactivated by incubation at 95 °C. Synthesised cDNA was utilised for analysis of gene expression profiles by quantitative RT-PCR. Quantitative RT primers were designed to target exon–exon boundaries. Detection of α-actin (*ACTA1*) was used as an internal control. For each sample, 10 μl (8 μl cDNA/SYBR green and 2 μl primer mix) was dispensed into three wells of a 384-well micro-amp plate (Applied Biosystems, Life Technologies Australia Pty Ltd., Scoresby, VIC, Australia) and amplified on an ABI 4900 light cycler. PCR conditions were 95 °C for 30 seconds, 60 °C for 30 seconds with 40 cycles and a final dissociation cycle. Analysis of amplification was performed after each cycle and again at the end of dissociation.

## Results

Using the GE INCA we have optimised a method of distinguishing the different major subtypes of primary human mammary epithelial cells (hMECs) in short-term primary culture. Cells were grown in multi-well plates for high-throughput power, the INCA was used for high-content fluorescent image acquisition and the IN Cell Developer Toolbox Software was used for automated digital image analysis. Fluorescent detection of K19 (far-red emission: false-coloured blue) and K14 (red emission) together delineated four possible subpopulations: K14^−^/K19^−^, DN; K14^+^/K19^−^, basal-like (K14^+^); K14^−^/K19^+^, luminal-like (K19^+^); and K14^+^/K19^+^, DP (Fig. 1c) – as reported previously [17]. To automate analysis of the immunofluorescent images we performed nuclear segmentation in the DAPI detection channel and measured the average fluorescence (density) of K14 and K19 within this defined area of the image (Fig. 1d). Plotting the natural logarithm of K14 and K19 pixel intensity for an average of 10,000 cells per sample produced the expected bimodal distributions within the mixed populations (Fig. 1e); on this mathematical basis, the thresholds for negative/low and positive expression were set per sample.

Assessment of cytokeratin staining in first-passage hMECs from five donors revealed considerable intra-donor and inter-donor heterogeneity in terms of subpopulation frequency (Fig. 1f). On average, the largest subpopulation was the exclusive K19^+^ subpopulation (40.6 %), followed by the exclusive K14^+^ (25.2 %) and DP (22.9 %), with DN cells comprising a minor subpopulation (11.3 %). Two of the five donors in this first analysis exhibited a skewed heterogeneity of subpopulations compared with the overall average. Interestingly, the youngest donor (Q626, 19 years old) exhibited a predominantly basal phenotype comprised of a significantly larger K14^+^ subpopulation compared with all other donors (*p* <0.001). In contrast, Q685 (44 years old) exhibited a predominantly luminal phenotype comprised of a significantly larger K19^+^ BP subpopulation compared with Q626 and Q695 (*p* <0.01). Our analyses of an additional five donors, however, did not reveal any statistically significant relationship between cell type frequency and donor age (Additional file 1: Figure S2) in this culture model.

To establish the robustness of our method we examined independent cultures from the same donors (Additional file 1: Figure S3A, B). Importantly we found that our culture method and assay were highly reproducible, calculating the same subpopulation frequencies from technical replicates of the plates. These data demonstrate that first-passage hMECs preserve a high degree of cellular heterogeneity and highlight the need to consider the mixture of cell types contained within the total population, rather than treating each donor sample as a phenotypically pure sample.

To demonstrate that our method could reliably detect changes in population phenotype, we assessed the effect of both passage and FACS-mediated enrichment for luminal-like and myoepithelial-like phenotypes on hMEC heterogeneity in each of these five donors. Enrichment for luminal-like and basal-like cells was performed by sorting for MUC1 and CD10 respectively in primary hMECs harvested after 7 days (P1), as described previously [18]. Sorted cells were cultured in parallel to ‘unsorted’ P2 cultures for a further 7 days before assessing K14 and K19 immunopositivity (Fig. 1g). We found few consistent changes in the four subpopulations amongst the donors with passage (P1 vs. P2), overall conserving a mixture of basal and luminal phenotypes. Interestingly, 3/5 donors showed a small but significant increase in DP cells at P2 (see Additional file 1: Figure S4). Others have indicated that passaging may select for a dominant myoepithelial phenotype within three passages [18], but in our hands luminal phenotypes are maintained at medium density second passage, suggesting that myoepithelial enrichment occurs with further passage. CD10^+^ enrichment produced consistent and significant enrichment of an average of 92 % K14^+^ cell subpopulations in all donors, with a concomitant decrease in K19^+^ cells (reduced to an average of 1 %) and DP (reduced to 2.8 %), indicating a strong basal-lineage purification and unipotency of CD10^+^ in terms of K14/K19 expression even after pre-sort and post-sort culture periods (Fig. 1g). In contrast, MUC1^+^ sorting produced more heterogeneous populations, retaining a significant proportion of cells with K14 positivity, albeit almost exclusively within the DP subpopulation, so that the progeny of MUC1^+^ are K19^+^ and DP subpopulations (averaging 54 % and 36 % respectively, with a minor K14^+^ population with a mean frequency of only 5.4 %). Other groups have similarly reported that a mixture of colony phenotypes is possible from MUC1^+^-sorted cells grown at clonal density [3, 18, 19].

**Fig. 2:**
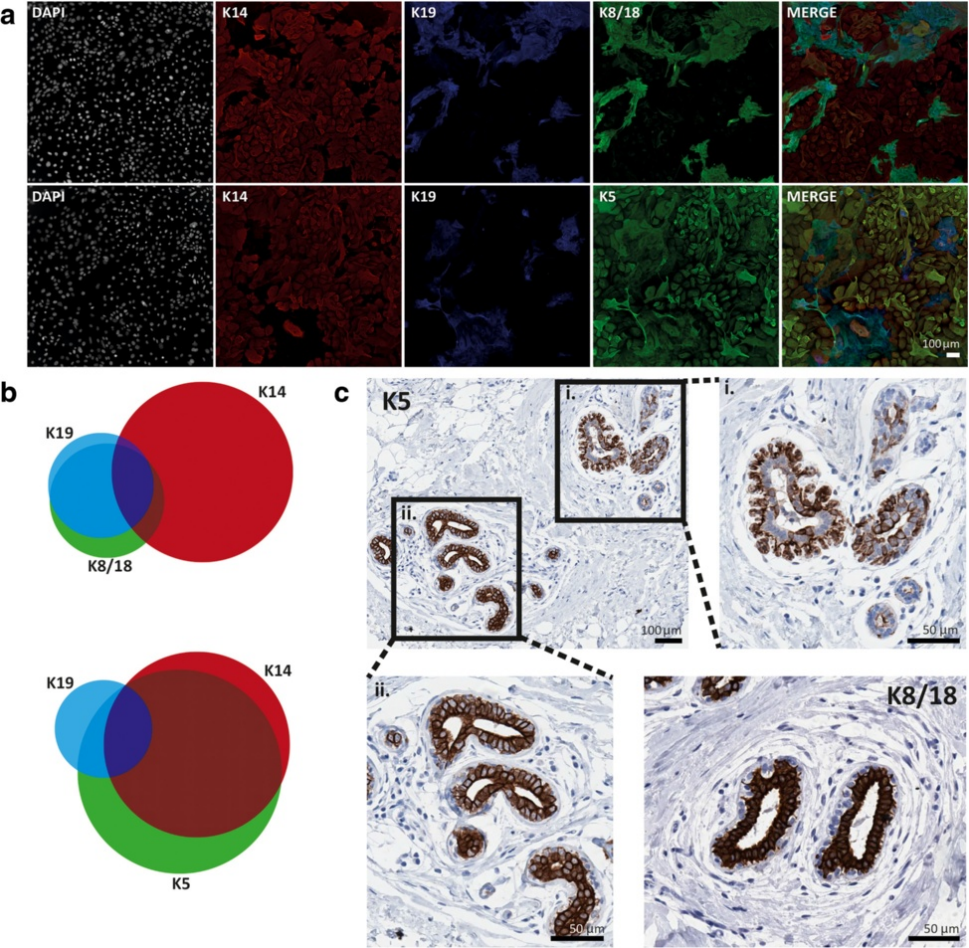
Assessment of alternative markers to delineate hMEC subpopulations using high-content screening. **a** Immunofluorescence staining of hMECs with DAPI (*white*) and antibodies against K14 (*red*), K19 (*blue*) and K8/18 (*green*; *top panel*) or K5 (*green*; *bottom panel*). Images acquired at 20× magnification using INCA. **b** Proportional Venn diagrams depict the mean overall proportions and overlap of K14, K8/18 and K19 expression (*top*) and K14, K5 and K19 expression (*bottom*) in first-passage hMEC cultures for donor Q626. **c** IHC staining of K5 and K8/18 on FFPE sections of normal human breast. Staining for K5 (*top left*, 10×) delineates both basal cells (i; *top right*, 20×) and luminal cells (ii; *bottom left*, 20×). Staining for K8/18 (*bottom right*, 10×) preferentially delineates basal cells. *DAPI* 4′,6-diamidino-2-phenylindole, *K5* cytokeratin 5, *K8/18* cytokeratin 8/18, *K14* cytokeratin 14, *K19* cytokeratin 19

Having established a reliable method for calculating K14/K19 frequencies, it was possible to exploit the additional capabilities of the INCA that allows image capture in up to eight channels. Cytokeratins 5 and 8/18 (K5 and K8/18) are also traditionally considered basal and luminal markers respectively, but their overlap with K14 and K19 in primary culture in vitro has not yet been quantitatively assessed. Simultaneous analysis of each of these two markers to an additional channel (Fig. 2a) yielded frequencies for a total of nine possible subpopulation combinations. Overall, K8/18 and K5 were more frequently expressed than K19 and K14 respectively in primary culture (Additional file 1: Figure S4). The K8/18-positive population overlapped very closely with that defined by the fellow luminal marker K19, as demonstrated by a Venn diagram representing subpopulation overlap for donor Q626 (Fig. 2b). The K5-defined subpopulation, however, comprised the majority of the total population, overlapping with large proportions of both the K19 and K14 defined subpopulations (Fig. 2b). In practical terms, this means K8/18 and K5 are less useful at delineating different in vitro subpopulations of hMECs, particularly regarding any myoepithelial-like and luminal-like dichotomy. Biologically, this is not surprising because both our immunohistochemistry (IHC) and others’ [20] highlights that, contrary to scientific opinion, K5 is by no means an exclusive basal/myoepithelial marker in vivo (Fig. 2c) [21]. Instead, even within the same small area of breast tissue, K5 can be found in both locations but more frequently associated with a luminal location, whilst K8/18, consistent with its overlap with K19 in vitro, is almost exclusively luminal (Fig. 2c).

**Fig. 3:**
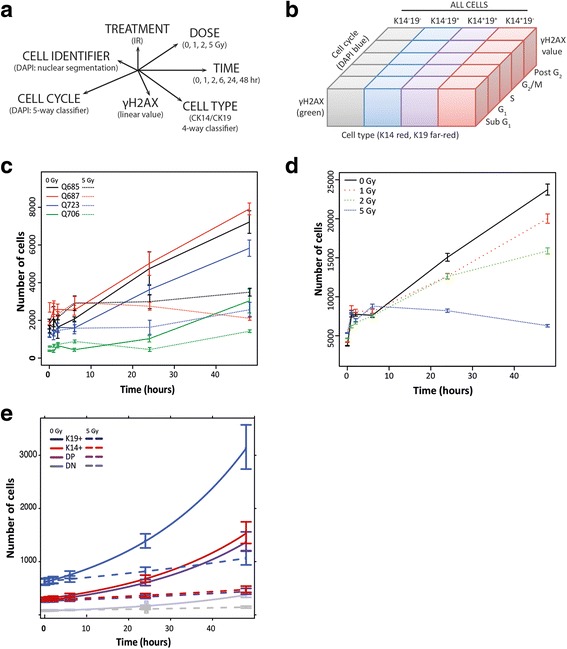
Growth pattern of hMECs with IR treatment and time. **a** Schematic representation of the multi-dimensional analysis performed with high-content screening. The different variables assessable by our experimental design are given, where axis length represents magnitude of variation and/or classification possible within the scope of this analysis. **b** Schematic representation of the variables simultaneously captured for a given population on a cell-by-cell basis using high-content screening. **c** Average number of cells by time and increasing IR dose for four donors. Mean total number of cells over (*n* = 5–6) technical replicates (± SEM) over 48 hours with 5 Gy IR treatment (*dashed line*) compared with controls (*solid line*, 0 Gy). **d** Response of hMECs to increasing dose of IR. Mean total number of cells (± SEM) over 48 hours with 0, 1, 2 and 5 Gy levels of IR for donor Q687. **e** Estimated growth pattern based on technical replicates of hMECs with time and dose stratified by cell phenotype or subpopulation. Estimated subpopulation (± SEM at fixed observation times) over 48 hours with and without treatment (5 and 0 Gy, respectively) plotted for donor Q687. *DAPI* 4′,6-diamidino-2-phenylindole, *DN,* double negative, *DP* double positive, *K14* cytokeratin 14, *K19* cytokeratin 19

Having established a reliable and precise method for discriminating between cell phenotypes in a mixed culture, we applied our assay to questions of cell-type specific DNA damage response. This high-content screening approach allows the flexibility to design and perform simultaneous multi-dimensional analyses that would otherwise be both too labour intensive, technically demanding and time consuming to perform with traditional manual approaches (Fig. 3a). The multi-factorial experimental design included the assessment of response to our chosen treatment (IR) with increasing dose over time. Four fluorescent channels were used for simultaneous analysis of four measurable variables: nuclear segmentation of DNA staining (DAPI) to identify cells allowing calculation of cell numbers; analysis of this same DNA intensity allowing analysis of cell cycle and quantification of frequencies in each cell cycle phase; analysis of nuclear γH2AX intensity, a marker of double-strand breaks, allowing measurement of response to DNA damage; and measurement of K14 and K19 intensity, as described, allowing all of these analyses to be performed with additional stratification by cell type. In this way, cells analysed at each dose and time point can be stratified by cell type and cell cycle phase and assessed for γH2AX expression (Fig. 3b).

Dose–response analyses of primary hMECs cultures from seven individual donors demonstrated that our high-content assay could faithfully detect the inhibition of total cell growth with radiation (Fig. 3c and Additional file 1: Figure S5A) within 48 hours in all donors, in a dose-dependent manner (Fig. 3d). Our method enabled stratification of growth response by cell type (Fig. 3e). To compare subpopulation-specific sensitivity to IR treatment, we assessed the proportions of the four cell types (K14^−^/K19^−^ (DN); K14^+^/K19^−^ basal-like (K14^+^); K14^−^/K19^+^ luminal-like (K19^+^); and K14^+^/K19^+^ (DP)) over time using split-plot analyses (Additional file 1: Figure S5B). As expected, we found no consistent significant differences across the seven individual donors. Of note, however, the baseline (untreated) population doubling time for hMECs revealed subpopulation differences in 3/7 donors (Q687, Q706 and Q767) where DP cells were significantly faster proliferators than the slower DN cells (Additional file 1: Figure S6), demonstrating that the starting population within each sample represents contrasting heterogeneity consistent with their possible identities as transitioning or transit-amplifying and quiescent stem cells respectively.

**Fig. 4:**
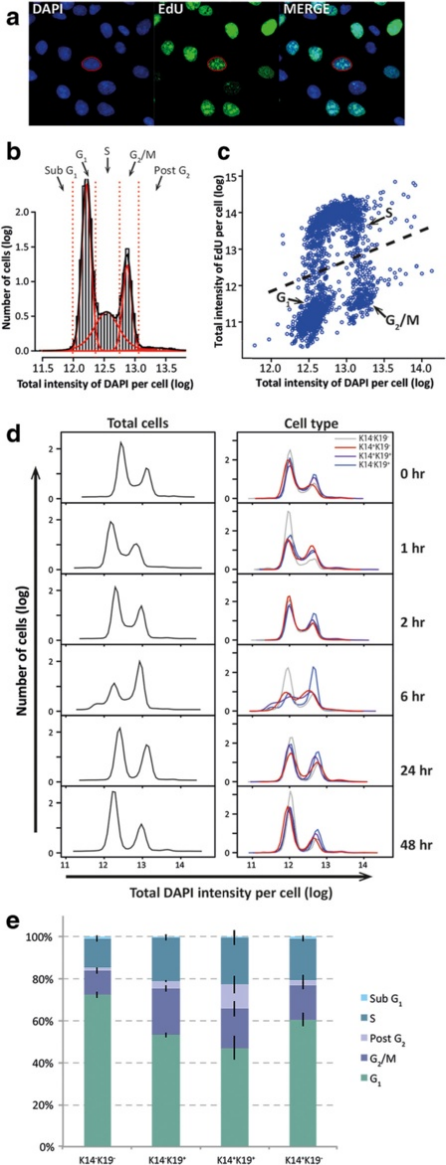
Cell cycle profile of hMECs with IR treatment and time. **a** Immunofluorescence staining of hMECs with DAPI (*blue*) for DNA content and the nucleoside analogue EdU (*green*) for newly synthesised DNA. **b** Representative distribution of total DAPI intensity for hMECs at baseline (0 Gy and 0 hours). The cell cycle phases are labelled, where the first peak represents cells with 2n DNA content (G_1_) and the second peak represents cells with 4n DNA content (G_2_/M). The distribution of total DAPI intensity is modelled using *t* distributions (*solid red lines*), and the intersection of these distributions (*dashed red lines*) are used to assign cell cycle phases. **c** Scatter plot of total EdU intensity against total DAPI intensity on a cell-by-cell basis. The relationship between the cell cycle phases (G_1_, S and G_2_/M) and total DAPI intensity (DNA content) vs. total EdU intensity (newly synthesised DNA) is indicated. **d** Mean proportion of cells within each cell cycle phase for each hMEC subpopulation at 0 Gy (± SEM). Cell cycle phase proportions from donor Q687. **e** Cell cycle phase distributions of hMECs post 5 Gy IR with time. Distributions of total DAPI intensity for all hMECs (*first column*) and stratified per hMEC subpopulation (*second column*) from donor Q685 post 5 Gy IR over 48 hours. *DAPI* 4′,6-diamidino-2-phenylindole

Our method also allows examination of cell cycle profile by high-content screening by plotting the distribution of total DNA staining intensity of the nuclear area (Fig. 4a, b). Analysis of cell cycle is a powerful mechanism to interpret cellular DNA damage status, cellular death, induction of arrest and mitotic aberrations. This method was further validated by simultaneous analysis of EdU incorporation to demonstrate new DNA synthesis detected from late G_1_, S to early G_2_/M (Fig. 4c). Stratification of cell cycle profiles by cell type revealed an increased G_1_ frequency in DN compared with other cell types (K19^+^, K14^+^, DP) in 3/4 donors (Fig. 4d, e and Additional file 1: Figure S7A, B), which is consistent with the population doubling times we observed for this cell type and the notion of a quiescent cell type.

**Fig. 5:**
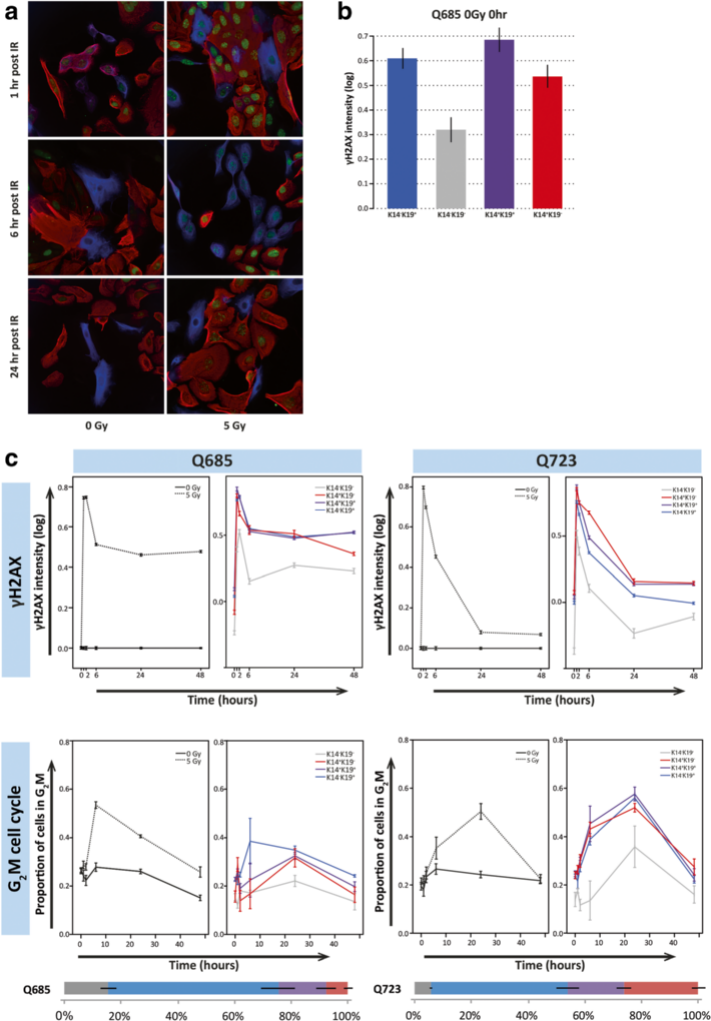
γH2AX response to IR in hMECs. **a** Representative immunofluorescent images of cells stained for K14 (*red*), K19 (*blue*) and γH2AX (*green*) at 1, 6 and 24 hours post 0 and 5 Gy IR. **b** Baseline levels of γH2AX in hMEC subpopulations. Mean total γH2AX intensity (log, ± SEM) for each hMEC subpopulation for donor Q685. **c** Effect of IR on γH2AX intensity and cell cycle profile in two donors with time. Mean total γH2AX intensity (log, ± SEM; *left panels*) and mean proportion of all cells within G_2_/M cell cycle phase (± SEM; *right panels*) for all cells at 0 Gy (*solid line*) and 5 Gy (*dashed line*) over time, and by stratified by cell type (*coloured*) plotted for 5 Gy treatment only over time, for donors Q685 (*top*) and Q723 (*bottom*). Baseline population phenotype heterogeneity is indicated as the frequency of hMEC subpopulations (± SEM) for both donors below the bottom panels. *IR* ionising radiation

Examination of cell cycle with time after treatment with 5 Gy IR revealed G_2_/M arrest that in two donors peaked after 6 hours (Fig. 4d) and in the other two donors at 24 hours. Our method was also able to stratify cell cycle phase by cell type and revealed that across all donors DN cells retain higher G_1_ frequencies than other cell types throughout the treatment period, suggestive of a resistance to the given treatment. Otherwise, despite cell profile indication of some donor-specific imbalances in cell cycle response, such as more profound G_2_/M arrest for K19^+^ cells at 6 hours post IR (Fig. 4d), overall observations did not achieve statistical significance (Fig. 5c).

To more specifically analyse the cellular response to DNA damage and repair kinetics, we added a fourth parameter to our simultaneous analysis. Phosphorylated H2AX (γH2AX) rapidly localises to foci of DNA double-stand breaks and its induction and resolution (upon repair) can be quantified by nuclear immunofluorescence using anti-γH2AX antibody (Fig. 5a). Interestingly, our analyses revealed a significantly lower baseline γH2AX level in the DN subpopulation in 5/7 donors examined (Fig. 5b), suggesting a more stable genome in this population. Examination of γH2AX in the total population after 5 Gy IR revealed two types of patterns amongst the seven donors analysed. In the first pattern, 5/7 donors exhibited a γH2AX peak at 1 hour and then resolved to close to baseline levels by 24–48 hours (Fig. 5c, Q723). Unexpectedly, we observed that two samples retained high γH2AX levels (Fig. 5c, Q685) for up to 48 hours. Interestingly, we observed that both donors with unresolved γH2AX levels are the same two donors demonstrating an earlier G_2_/M peak at 6 hours compared with 24 hours in the donors whose γH2AX resolves. These differences in response kinetics could have several explanations. The donors with earlier G_2_/M peak may arrest earlier but inefficiently, unable to maintain arrest and repair DNA damage and resolve γH2AX. The prolonged arrest at 24 hours might also suggest residual damage, blocked cytokinesis from the genotoxic insult or perhaps even impaired S-phase checkpoints [22]. It is also possible that inter-individual differences exist in the use of different DNA damage repair mechanisms such as non-homologous end-joining and homologous recombination; however, this would require further investigation.

Our screen for cell-type specific analyses revealed some donor-specific differences but gave varied results across donors in terms of both cell cycle arrest and γH2AX induction and resolution (Additional file 1: Figure S8). Although no consistent subpopulation-specific changes were observed across the donor cohort (Additional file 1: Figure S5B), closer interrogation of these data revealed that the two donors exhibiting sustained γH2AX and an earlier G_2_/M arrest described earlier show a significant change (reduction) in K19^+^ subpopulation frequency in response to radiation treatment over time and a concomitant increase in DP cells (Additional file 1: Figure S5B). It could be that DP cells represent those with enhanced genome instability and sustained DNA damage in response to IR. Whilst this analysis has revealed some interesting differential responses to DNA damage, screening of a larger cohort in which we could better control for several parameters of inter-individual difference such as age (which may in turn affect), population doubling time, baseline cell cycle profile and baseline cell-type heterogeneity is necessary to determine which consistent differences merit further and more specific cellular and molecular investigation.

**Fig. 6:**
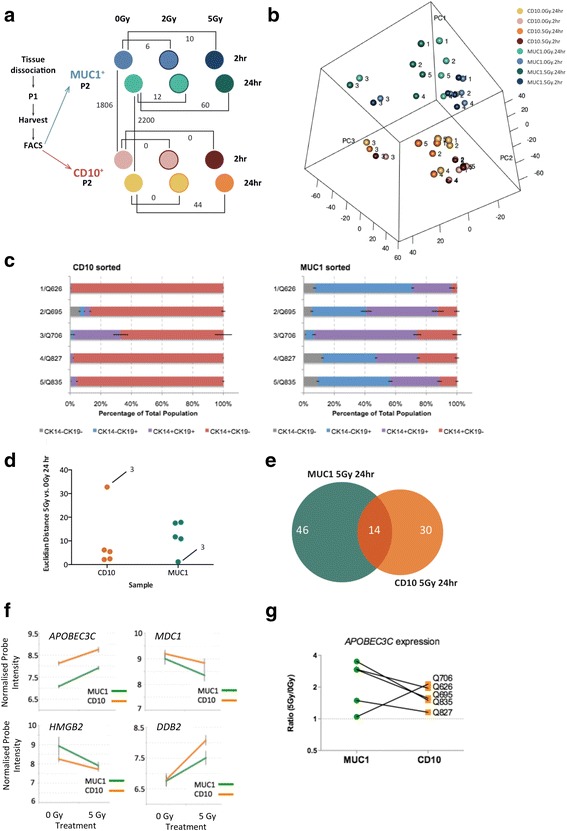
Differential gene expression analysis of hMEC subpopulations treated with IR. **a** Experimental design to determine genes differentially expressed between CD10^+^ and MUC1^+^ enriched cultures (matched per donor) treated with IR. Freshly dissociated hMECs were cultured in vitro for 7 days (P1), then harvested and sorted based on expression of CD10 and MUC1 using FACS. Sorted cells were cultured for 5 days (P2), treated with 0, 2 and 5 Gy IR and then collected 2 and 24 hours post IR. RNA from all 12 samples was extracted and subjected to microarray analysis. The number of differentially expressed genes between the samples is indicated. **b** Principal component analysis of the expression of 19,472 filtered probes. Control (0 Gy) and 5 Gy treated samples of donor-matched (*n* = 5) CD10 and MUC1 sorted cultures are plotted. For clarity, donors are annotated consecutively: Q626, *1*; Q695, *2*; Q706, *3*; Q827, *4*; Q835, *5*. **c** Subpopulation frequencies within hMEC cultures from MUC1^+^-sorted and CD10^+^-sorted cells performed in parallel to gene expression profiling. Mean subpopulation frequencies were calculated as the average percentage of total cell number per technical replicate for each sample (± SEM). DP frequencies of Q706 in CD10-sorted cells and MUC1-sorted cell K19^+^ frequencies were statistically different from all other donors (*p* <0.0001). **d** Dot plot demonstrating the Euclidian distance measured between 0 and 5 Gy matched samples at 24 hours of MUC1-sorted and CD10-sorted cultures. **e** Proportional Venn diagram depicting the number of shared and exclusive differentially expressed genes between MUC1 and CD10 samples 24 hours post 5 Gy IR treatment. **f** Normalised probe intensity from the gene expression analysis comparing CD10 and MUC1 samples at 24 hours after 5 Gy or no (0 Gy) IR treatment shown as the mean of five donors. **g** Relative expression of *APOBEC3C* in MUC1 and CD10 cultures before and after IR treatment shown as fold-change. The ratio of 5 Gy:0 Gy was significantly >1 for MUC1 (*p* = 0.034), whereas for CD10 the ratio was not significantly different from 1 (*p* = 0.84). *FACS* fluorescence-activated cell sorting, *P1* passage 1, *P2* passage 2

As a differential response to IR may not be limited to our detection of these parameters, another approach was needed to make a more discovery-based assessment as to whether global changes may be occurring in these different subpopulations, possibly before an overt phenotypic change. A more global approach, such as gene expression profiling, is in turn limited by the amount of RNA required, and thus primary cultures from pre-sorted cells enriched for the particular K14/K19 phenotypes were profiled. In order to observe the molecular differences in the different cell subpopulation response to IR, we also performed gene expression profiling of MUC1^+^-sorted and CD10^+^-sorted primary hMEC cultures (subsequently referred to as MUC1 cultures and CD10 cultures) which we had previously shown to be enriched for K19^+^/DP and K14^+^ cell subtypes respectively and quantified here in a parallel assay. Transcriptional response was measured at 2 and 24 hours after treatment with 2 and 5 Gy IR via expression array (Fig. 6a). The complete sample cohort included time point matched, untreated (0 Gy) controls in a total of five individual donors. Comparison between the MUC1 and CD10 culture baseline (untreated) controls revealed differential expression of 1806 and 2200 probes at 2 and 24 hours respectively (Additional file 1: Figure S9A) and included many expected luminal and myoepithelial markers based on previous studies [23], including greater expression of *MUC1* itself, *KRT8* (K8) and *CLDN4* (Claudin 4) in MUC1 cultures, with *ITGA6* (a6 integrin), *TP63*, *VIM* (vimentin) and *NRG1* (neuregulin 1) more highly expressed in CD10 cultures. Interestingly we found higher levels of *PROM1* (prominin 1; CD133) and *ALDH3B1* (aldehyde dehydrogenase 3 family member B1) in the MUC1 cultures, which are both associated with stem-like and progenitor-like phenotypes [1, 24, 25] (Additional file 1: Figure S9B). Increased expression of established luminal marker *KRT19* in MUC1 compared with CD10 was revealed by the arrays, validating in 5/5 donors by quantitative PCR (*p* <0.05; data not shown), and also notably confirmed at the protein level with increased frequency of K19^+^ subpopulations (Fig. 6c).

Unsupervised PCA of the 19,472 filtered probes illustrated the overall transcriptional differences between all samples (Fig. 6b). This rotation matrix (and dendrogram generated by unsupervised hierarchical clustering in Additional file 1: Figure S9B) indicates that the samples group, firstly, according to their sort population origin (MUC1^+^ and CD10^+^; proportion of variance 33.4 %) and, secondly, according to time point (2 and 24 hours; proportion of variance 26.7 %). The remaining components demonstrate negligible amounts of the variability within the data (<5 %) and confirm INCA observations that cellular attributes cluster according to individual donor irrespective of treatment.

Interestingly, one donor (3/Q706, 46 years old) clustered away from all others for both MUC1-sorted and CD10-sorted cultures. Our INCA method performed in parallel was able to provide some explanation for these differences by quantifying the heterogeneity that still exists within the enriched but still mixed primary cultures of sorted cells. Compared with other donors, Q706 exhibited significantly more CK19-positivity and more CK14-positivity in CD10 and MUC1 cultures respectively (Fig. 6c); indeed the dendrogram resulting from the unsupervised hierarchical cluster analysis shows that some of the CD10 cultures of this donor clustering together with its MUC1 counterparts (Additional file 1: Figure S9B). The analytical power of our fluorescence-based method is able to confirm phenotypic heterogeneity of primary cultures providing insight into potential genetic variation.

Despite the strong donor effect, we do observe that 5 Gy treatment yields a greater overall transcriptional response and variation in gene expression change in the MUC1 cultures compared with the CD10 cultures at 24 hours shown by comparing the Euclidian distance between the matched samples (Fig. 6d). Q706 is also shown to be an outlier in this comparison; its omission (on the basis of general phenotypic differences from other donors) finds the difference between MUC1 and CD10 cultures’ IR-induced change to be statistically significant (*p* = 0.035). Consistent with this, MUC1 cultures exhibited differential gene expression at earlier time points (2 hours) and to lower dose of radiation (2 Gy) whilst CD10 did not (Fig. 6a), suggesting that MUC1 cultures are compensating for increased sensitivity to DNA damage. The greatest transcriptional response in both culture types was observed 24 hours after 5 Gy irradiation, when MUC1 cultures showed differential expression of 60 genes whilst CD10 revealed only 44, 14 of which were common to both cultures (Fig. 6e and Additional file 2).

Ingenuity pathway analysis of the 5 Gy 24-hour response revealed, as expected, overrepresentation of cell cycle, of DNA replication, recombination and repair, and of cell death and survival pathways in the same order of significance in both MUC1 and CD10 cultures (Additional file 1: Table S1). The transcriptional data for both MUC1 and CD10 cultures indicated, as expected, upstream activation of TP53 evidenced by upregulation of several common genes including *TRIM22*, *SPATA18*, *FAS*, *DDB2*, *RRM2B*, *BAX* and *CCNG1*, as well as upregulated *CDKN1A*, *BTG2*, *ACTA2*, *CDCA4*, *TP53AP1* and downregulated *CDC25C*, *KIF15*, *HMGB2*, *MDC1*, *CDCA4* and *SMC2* exclusive to MUC1 and upregulated *ANXA4* and *RAD51C* exclusive to CD10. We technically validated these changes to IR in DNA damage responsive genes *DDB2*, *HMGB2* and *MDC1* by quantitative RT-PCR, which correlated with the array data showing *DDB2* upregulated with IR in all but one sample of both subpopulations from five donors, while *HMGB2* and *MDC1* are consistently downregulated (Fig. 6f and Additional file 1: Figure S10).

The most highly differentially altered genes exclusive to MUC1 cultures were *ACTA2* (aortic smooth muscle actin) and *CDKN1A* (p21; cyclin-dependent kinase inhibitor 1A) and to CD10 cultures were *KITLG* (KIT ligand) and *CYFIP2* (cytoplasmic FMR1 interacting protein 2). Of the 14 IR-responsive genes common to both culture types, four were also differentially expressed between MUC1 and CD10 cultures at baseline (Fig. 6f and Additional file 2): E3 ubiquitin-protein ligase *TRIM22* (tripartite motif containing 22), the DNA deaminase *APOBEC3C* (apolipoprotein B mRNA editing enzyme, catalytic polypeptide-like 3C), the death receptor FAS (TNF receptor superfamily, member 6) and *C7ORF10*. Whilst endogenous *APOBEC3C* levels were higher in CD10 cultures, induction upon irradiation was shown to be greater in MUC1 cultures (Fig. 6f). Quantitative PCR validation revealed 3/5 donors show a statistically significant (*p* <0.05) increase in *APOBEC3C* in CD10 versus MUC1 cultures at baseline, and a greater IR-induced change in MUC1 versus CD10 pairs in 4/5 donors (*p* <0.05; Fig. 6g). The potential importance of this finding lies in an International Cancer Genome Consortium study which recently provided evidence that aberrant function of this class of enzymes is responsible for specific mutational signatures of cancer, including two different signatures found in breast cancer [26].

## Discussion

We present a high-throughput, high-content fluorescence microscopy approach for screening cell-type specific effects in mixed primary breast epithelial culture. The principle of this method can be applied to any mixed culture method, including co-cultures and mixed primary tissues, where reliable strong cytoplasmic or nuclear markers for differentiating cell types are available. The digital analysis of alternative membrane (as used in flow cytometry) or cytoplasmic markers using digital segmentation of these defined subcellular areas (*i.e.* cell membrane, cell cytoplasm or whole cell) was found to harbour significant limitations, particularly in assigning expression to individual cells due to the tight cell–cell interactions and cobblestone morphology of epithelial cell culture, and was cumbersome and costly so was precluded from further use. We found instead, as shown, that analysis of abundant and easily detected cytoskeletal proteins (in this instance optimised for four different cytokeratins), albeit in that area of the captured images defined by the nucleus (and therefore a detection of fluorescence from cytokeratins in planes above and below the nucleus), produced the expected bimodal distribution of a primary breast culture with the additional advantage of perfect correlation with cell/nuclear count. Whilst our approach may be limited to targets in the nuclear plane, this itself is a solution to a common scenario wherein membrane or (greater) cytoplasmic detection is problematic and in itself limiting the use of this very powerful technology. We believe we have now made available to the field of normal breast biology a very powerful method to assay cell-type specific biology using the K14/K19 stratification upon which detection of other proteins or assessment of other behaviour can now be overlaid and quantitatively measured. It would be very interesting, for instance, to use this method to quantify the effect of different culture media, matrix proteins and growth factors on the frequency and growth of different phenotypes and also to track such changes closely with time. It is also feasible that the assay could be adapted to include assessment of colonies to measure progenitor activity and also slides of smeared or cytospun cells to assess phenotype prior to culture. Importantly, the principles applied in our method have much broader application than our tissue of interest and could, for example, include a similar use of cytokeratins for distinguishing various subtypes of epidermal cells, vimentin for the detection of mesenchymal or stromal cells, GFAP (Glial fibrillary acidic protein) for neural cells or an even more extensive detection of exogenous label such as whole cell fluorescent stains in co-cultures or GFP (Green Fluorescent Protein)-labelled targets, and is certainly also applicable to cell culture derived from animal models.

Of further note, we found that despite optimising and saturating antibody concentrations and high reproducibility shown in technical replicates, in a single multi-well plate the distribution of cytokeratin intensity could demonstrate small shifts between samples either from different donors, time points or cell densities. Although these differences were subtle and would not be detectable using traditional manual scoring or assessment of a small number of cells, we were able to see that application of a single threshold to an entire plate of samples would lead to imperfect classification of cell types. We were able to overcome this by calculating mathematical thresholds for each sample that defined a positive sample. We also determined that the optimal method for calculation of this involved application of multiple Gaussian distributions (comprised of both the stained and unstained population) and then selection of the first intersection of the distribution which occurred after the sample-specific secondary only population arrives at zero. This is an important consideration for others optimising the assessment of similar parameters in high-throughput format.

K14 and K19 have been most notably studied by Villadsen et al. [17] to delineate four normal breast epithelial subpopulations both in vivo and in vitro. This same group suggested that the DP cells may represent a transitioning cell and are found as single scattered or small groups of cells in the ducts. Our data showing increasing frequencies of the DP cells with passage and with MUC1 sorting and co-expression with K5 may suggest that in the primary culture setting these cells are transitioning ‘luminal-like’ or ‘luminal-progenitor-like’ cells. In our experiments we may be observing either a selection and expansion of luminal progenitor cells or these markers are induced with primary culture. It is possible that such cells are actually de-differentiating from the luminal compartment. Our findings, together with a recent demonstration that myoepithelial cells express CD10 in situ [20], suggest that DP cells are luminal with basal features and demonstrate a greater plasticity of this compartment in these culture conditions. In vivo, the DN cells were rare clones found within the lobules consistent with quiescent stem cell phenotype [17]. Consistent with this, in our experiments we found that frequencies were always minor and constant, relatively unperturbed by time or treatment. They had a high frequency of cells in G_1_ and exhibited low baseline γH2AX suggestive of both quiescence and resistance to DNA damage respectively. Further investigations of this cell type are needed, as the possible although unlikely contribution of stromal contaminants cannot be entirely excluded without additional markers; however, our assay provided an excellent platform upon which the different functional biologies of these different cell types could be explored.

Comprehensive investigation of the environmental contribution to the aetiology of breast cancer reveals that IR (together with hormone therapy) bears the strongest association to development of the disease [27]. Natural background radiation is very low (approximately 0.27 mGy per year) but together with exposure from man-made sources, including increasingly diffuse medical imaging (8.0 mGy for abdominal CT) and radiation therapy (up to 60 Gy divided into smaller fractions for solid tumours), contributes to an individual’s lifetime exposure, in addition to unfortunate acute events which carry higher risk of cancer development. An individual’s age at exposure, genetic profile and possibly oestrogen levels may all interact with radiation to increase the potency of its carcinogenic effect in a highly complex relationship. Cells immediately activate a series of biochemical pathways in response to IR that promote cell survival while maintaining genetic integrity. We hypothesise that the different subpopulations of normal breast epithelial cells might have different sensitivity and responses to such damage, which might underlie the carcinogenesis of particular breast cancer subtypes. Of the handful of studies aimed at addressing similar biological questions, Huper and Marks [28] demonstrated that basal cells undergo a rapid but labile cell cycle arrest that may underlie their increased susceptibility to transformation, whereas luminal cells show a much more durable arrest, primarily at the G_2_/M boundary. In contrast, we could not with any consistency reproduce this effect in our larger cohort, possibly explained by differences in experimental design. We and others have shown that FACS-sorted cultures (as used by Huper and Marks [28]) are not homogeneous and re-equilibrate their heterogeneity with time in culture [29, 30]. Our assessment of cell-type cell cycle response was, instead, performed with stratification of response by cell type in mixed culture. Furthermore, the two separate enriched cultures compared with each other by Huper and Marks [28] were grown in different appropriate media whilst our assay has the advantage of simultaneously assessing cell-type specific response in mixed culture (*i.e.* the same environment and culture conditions). Mixed primary culture more faithfully recapitulates the interactions of the in vivo environment over the enriched populations that are produced with sorting.

Lastly, the advent of a high-throughput assay such as this permits the analysis of an increased number of cells and therefore the generation of greater and multifaceted data from donor samples. This is a particularly important advantage for the study of cells derived from access-limited specimen such as reduction mammoplasty specimens. There is a paucity of normal breast tissue donor cohorts, and previous studies of this kind include a largely qualitative account of response in (at most) 10 patient samples [31] and another restricted cohort including a single reduction mammoplasty sample examined by IHC [28], making our highly quantitative approach, using multiple technical and biological replicates, a large advance on these previous accounts. The small donor numbers are usually balanced, however, by the greater physiological relevance of primary human breast tissue data over that produced by cell lines and mouse tissue. Nonetheless, further detailed investigation is needed to identify the different DNA damage response mechanisms that may be differentially active between the different cell subtypes and donors, and also to determine whether the pattern of outlier behaviour is observable in specimens from additional donors and represents a subgroup of individuals. The data from this initial screen make the exciting suggestion, however, that differences exist between the normal breast epithelium of healthy donors and may be very important in predisposition to breast cancer.

Intriguingly, our gene expression analysis of the different normal breast cellular compartments revealed an increased expression of *APOBEC3C* in MUC1^+^ luminal-like sorted cells in response to IR. Whilst our cohort is limited in number and independent validation on a greater number of donor samples is needed, a role for this gene is further suggested by the detection of its differential expression even at both 5 Gy and lower dose (2 Gy) in the MUC-1-sorted cells (Additional file 2). The APOBEC family of cytidine deaminases has recently been shown to account for two signatures of localised substitution hypermutation termed ‘kaetegis’ [26]. To date, only a small number of studies have investigated the role of the APOBEC family of enzymes in breast cancer tumourigenesis [32, 33], but indicate that over-activity of these enzymes can cause cell cycle deviation, DNA fragmentation and γH2AX accumulation, and C to T mutation implicating a chronic source of DNA damage that may select for *TP53* inactivation [34]. Furthermore, germline polymorphisms in the *APOBEC3* cluster genes have been associated with breast cancer susceptibility [35]. Interestingly, tumours of these donors show increased activity of APOBEC-dependent mutational processes, although mechanistic details by which deletions are introduced remain unknown [32]. In that regard, expression of APOBEC family members in yeast can recapitulate cancer-like kaetegis, particularly in the vicinity of induced double-strand breaks [36]. The same study also demonstrated through comparison of mutation spectra that APOBEC3B and/or APOBEC3A were most likely responsible for the breast cancer hypermutation patterns, but allowed the possibility that other APOBEC3s might also contribute to genome mutation. Our study adds some very interesting insight into the possible mechanism by which APOBEC family members might contribute to cancer initiation in the breast, by uncovering the consistent upregulation of *APOBEC3C* after genotoxic insult in normal breast epithelial cells and its differential baseline expression in normal luminal-like and basal-like cells. It is possible that an imbalance in the activity of the different APOBEC family cytidine deaminases underlies the generation of kaetegis. Further studies are required to fully elucidate the role of APOBEC-mediated breast cancer tumourigenesis; however, in a cell-type specific context using an assay such as ours, there is merit in marrying this new technique of investigation for APOBEC-mediated mutational signatures of breast cancer cells with the idea of defining a specific cell of origin for different breast cancer subtypes.

## Conclusion

Clearly, as seen in our study of 12 donors, the role of individual biology is a significant contributing factor to data interpretation, and therefore high-throughput methods that facilitate the analysis of larger sample cohorts are very much needed. We believe that this assay represents a new and powerful tool for high-throughput screening of primary normal human breast epithelial cultures. Further, the assay offers quality outputs of easy interpretation with mathematically delineated parameters that can be manipulated to effectively address complex, untested biological questions in many fields of research where mixed, particularly primary, culture models are available.

## Additional files

Additional file 1:
**contains Figures S1–S10 and Table S1 (ingenuity pathway analysis).** (PDF 3118 kb)
Additional file 2:
**contains Table S2 (differentially expressed genes by group, and genes by sample).** (XLSX 156 kb)

## Abbreviations

ANOVA: Analysis of variance
DAPI: 4′,6-Diamidino-2-phenylindole
DN: Double negative
DP: Double positive
FACS: Fluorescence-activated cell sorting
FBS: Foetal bovine serum
FITC: Fluorescein isothiocyanate
HBSS: Hank’s Balanced Salt Solution
hMEC: Human mammary epithelial cell
IHC: Immunohistochemistry
INCA: IN Cell Analyser 2000
IR: Ionising radiation
K5: Cytokeratin 5
K8/18: Cytokeratin 8/18
K14: Cytokeratin 14
K19: Cytokeratin 19
P1: Passage 1
P2: Passage 2
PBS: Phosphate-buffered saline
PCA: Principal components analysis
PE: Phycoerythrin
RT: Reverse transcriptase
SEM: Standard error of the mean

## References

[1] Lim E, Vaillant F, Wu D, Forrest NC, Pal B, Hart AH (2009). Aberrant luminal progenitors as the candidate target population for basal tumor development in BRCA1 mutation carriers. Nat Med..

[2] Keller PJ, Arendt LM, Skibinski A, Logvinenko T, Klebba I, Dong S (2012). Defining the cellular precursors to human breast cancer. Proc Nat Acad Sci U S A..

[3] Raouf A, Zhao Y, To K, Stingl J, Delaney A, Barbara M (2008). Transcriptome analysis of the normal human mammary cell commitment and differentiation process. Cell Stem Cell..

[4] Molyneux G, Geyer FC, Magnay FA, McCarthy A, Kendrick H, Natrajan R (2010). BRCA1 basal-like breast cancers originate from luminal epithelial progenitors and not from basal stem cells. Cell Stem Cell..

[5] Keller PJ, Lin AF, Arendt LM, Klebba I, Jones AD, Rudnick JA (2010). Mapping the cellular and molecular heterogeneity of normal and malignant breast tissues and cultured cell lines. Breast Cancer Res..

[6] Lim E, Wu D, Pal B, Bouras T, Asselin-Labat ML, Vaillant F (2010). Transcriptome analyses of mouse and human mammary cell subpopulations reveal multiple conserved genes and pathways. Breast Cancer Res..

[7] Proia TA, Keller PJ, Gupta PB, Klebba I, Jones AD, Sedic M (2011). Genetic predisposition directs breast cancer phenotype by dictating progenitor cell fate. Cell Stem Cell..

[8] Kannan N, Huda N, Tu L, Droumeva R, Aubert G, Chavez E (2013). The luminal progenitor compartment of the normal human mammary gland constitutes a unique site of telomere dysfunction. Stem Cell Reports..

[9] Clarke CL, Sandle J, Jones AA, Sofronis A, Patani NR, Lakhani SR (2006). Mapping loss of heterozygosity in normal human breast cells from BRCA1/2 carriers. Br J Cancer..

[10] Lakhani SR, Chaggar R, Davies S, Jones C, Collins N, Odel C (1999). Genetic alterations in 'normal' luminal and myoepithelial cells of the breast. J Pathol..

[11] Smart CE, Morrison BJ, Saunus JM, Vargas AC, Keith P, Reid L (2013). In vitro analysis of breast cancer cell line tumourspheres and primary human breast epithelia mammospheres demonstrates inter- and intrasphere heterogeneity. PLoS One..

[12] Stingl J, Eaves CJ, Emerman JT, Ip MM, Asch BB (2000). Characterization of normal human breast epithelial cell subpopulations isolated by fluorescence-activated cell sorting and their clonogenic growth *in vitro*. Methods in mammary gland biology and breast cancer research.

[13] Ethier SP, Mahacek ML, Gullick WJ, Frank TS, Weber BL (1993). Differential isolation of normal luminal mammary epithelial cells and breast cancer cells from primary and metastatic sites using selective media. Cancer Res..

[14] Efron B, Tibshirani R, Storey JD, Tusher V (2001). Empirical Bayes analysis of a microarray experiment. J Am Stat Assoc..

[15] Smyth GK, Gentleman R, Carey V, Dudoit S, Irizarry R, Huber W (2005). Limma: linear models for microarray data. Bioinformatics and computational biology solutions using R and bioconductor.

[16] Smyth GK (2004). Linear models and empirical bayes methods for assessing differential expression in microarray experiments. Stat Appl Genet Mol Biol.

[17] Villadsen R, Fridriksdottir AJ, Ronnov-Jessen L, Gudjonsson T, Rank F, LaBarge MA (2007). Evidence for a stem cell hierarchy in the adult human breast. J Cell Biol..

[18] Stingl J, Eaves CJ, Kuusk U, Emerman JT (1998). Phenotypic and functional characterization in vitro of a multipotent epithelial cell present in the normal adult human breast. Differentiation..

[19] Pechoux C, Gudjonsson T, Ronnov-Jessen L, Bissell MJ, Petersen OW (1999). Human mammary luminal epithelial cells contain progenitors to myoepithelial cells. Dev Biol..

[20] Santagata S, Thakkar A, Ergonul A, Wang B, Woo T, Hu R (2014). Taxonomy of breast cancer based on normal cell phenotype predicts outcome. J Clin Invest..

[21] Gusterson BA, Ross DT, Heath VJ, Stein T (2005). Basal cytokeratins and their relationship to the cellular origin and functional classification of breast cancer. Breast Cancer Res.

[22] Xu B, Kim ST, Lim DS, Kastan MB (2002). Two molecularly distinct G(2)/M checkpoints are induced by ionizing irradiation. Mol Cell Biol..

[23] Jones C, Mackay A, Grigoriadis A, Cossu A, Reis-Filho JS, Fulford L (2004). Expression profiling of purified normal human luminal and myoepithelial breast cells: identification of novel prognostic markers for breast cancer. Cancer Res..

[24] Choudhury S, Almendro V, Merino VF, Wu Z, Maruyama R, Su Y (2013). Molecular profiling of human mammary gland links breast cancer risk to a p27(+) cell population with progenitor characteristics. Cell Stem Cell..

[25] Eirew P, Kannan N, Knapp DJ, Vaillant F, Emerman JT, Lindeman GJ (2012). Aldehyde dehydrogenase activity is a biomarker of primitive normal human mammary luminal cells. Stem Cells..

[26] Alexandrov LB, Nik-Zainal S, Wedge DC, Aparicio SA, Behjati S, Biankin AV (2013). Signatures of mutational processes in human cancer. Nature..

[27] Smith-Bindman R (2012). Environmental causes of breast cancer and radiation from medical imaging: findings from the Institute of Medicine report. Arch Intern Med..

[28] Huper G, Marks JR (2007). Isogenic normal basal and luminal mammary epithelial isolated by a novel method show a differential response to ionizing radiation. Cancer Res..

[29] Fillmore CM, Kuperwasser C (2008). Human breast cancer cell lines contain stem-like cells that self-renew, give rise to phenotypically diverse progeny and survive chemotherapy. Breast Cancer Res..

[30] Gupta PB, Fillmore CM, Jiang G, Shapira SD, Tao K, Kuperwasser C (2011). Stochastic state transitions give rise to phenotypic equilibrium in populations of cancer cells. Cell..

[31] Coates PJ, Appleyard MV, Murray K, Ackland C, Gardner J, Brown DC (2010). Differential contextual responses of normal human breast epithelium to ionizing radiation in a mouse xenograft model. Cancer Res..

[32] Nik-Zainal S, Wedge DC, Alexandrov LB, Petljak M, Butler AP, Bolli N (2014). Association of a germline copy number polymorphism of APOBEC3A and APOBEC3B with burden of putative APOBEC-dependent mutations in breast cancer. Nat Genet..

[33] Burns MB, Lackey L, Carpenter MA, Rathore A, Land AM, Leonard B (2013). APOBEC3B is an enzymatic source of mutation in breast cancer. Nature..

[34] Waters CE, Saldivar JC, Amin ZA, Schrock MS, Huebner K (2014). FHIT loss-induced DNA damage creates optimal APOBEC substrates: Insights into APOBEC-mediated mutagenesis. Oncotarget.

[35] Long J, Delahanty RJ, Li G, Gao YT, Lu W, Cai Q (2013). A common deletion in the APOBEC3 genes and breast cancer risk. J Natl Cancer Inst..

[36] Taylor BJ, Nik-Zainal S, Wu YL, Stebbings LA, Raine K, Campbell PJ (2013). DNA deaminases induce break-associated mutation showers with implication of APOBEC3B and 3A in breast cancer kataegis. eLife..

