# Supplementary material for: High content screening application for cell-type specific behaviour in heterogeneous primary breast epithelial subpopulations
Source: Breast Cancer Res. 2016 Feb 9;18:18. doi: 10.1186/s13058-016-0681-9 (PMC4748588; doi:10.1186/s13058-016-0681-9)
Supplement: Additional file 1: — contains Figures S1–S10 and Table S1 (ingenuity pathway analysis). (PDF 3118 kb) [file 13058_2016_681_MOESM1_ESM.pdf]

Supplementary Figure 1

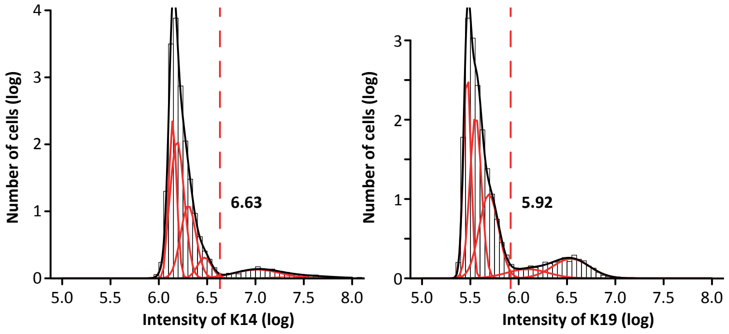

Supplementary Figure 2

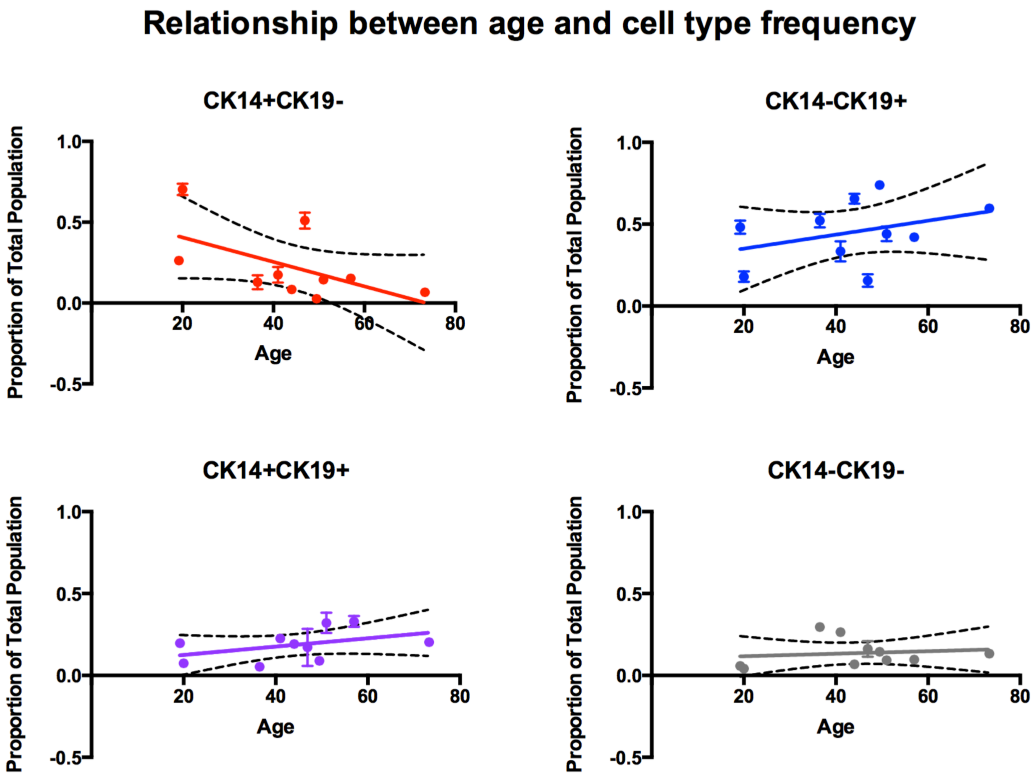

Supplementary Figure 3

A

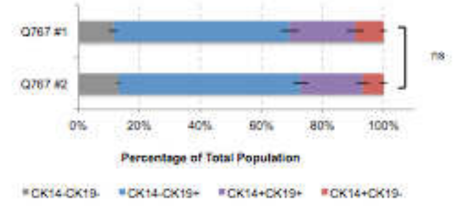

B

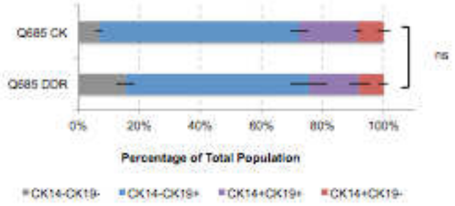

Supplementary Figure 4

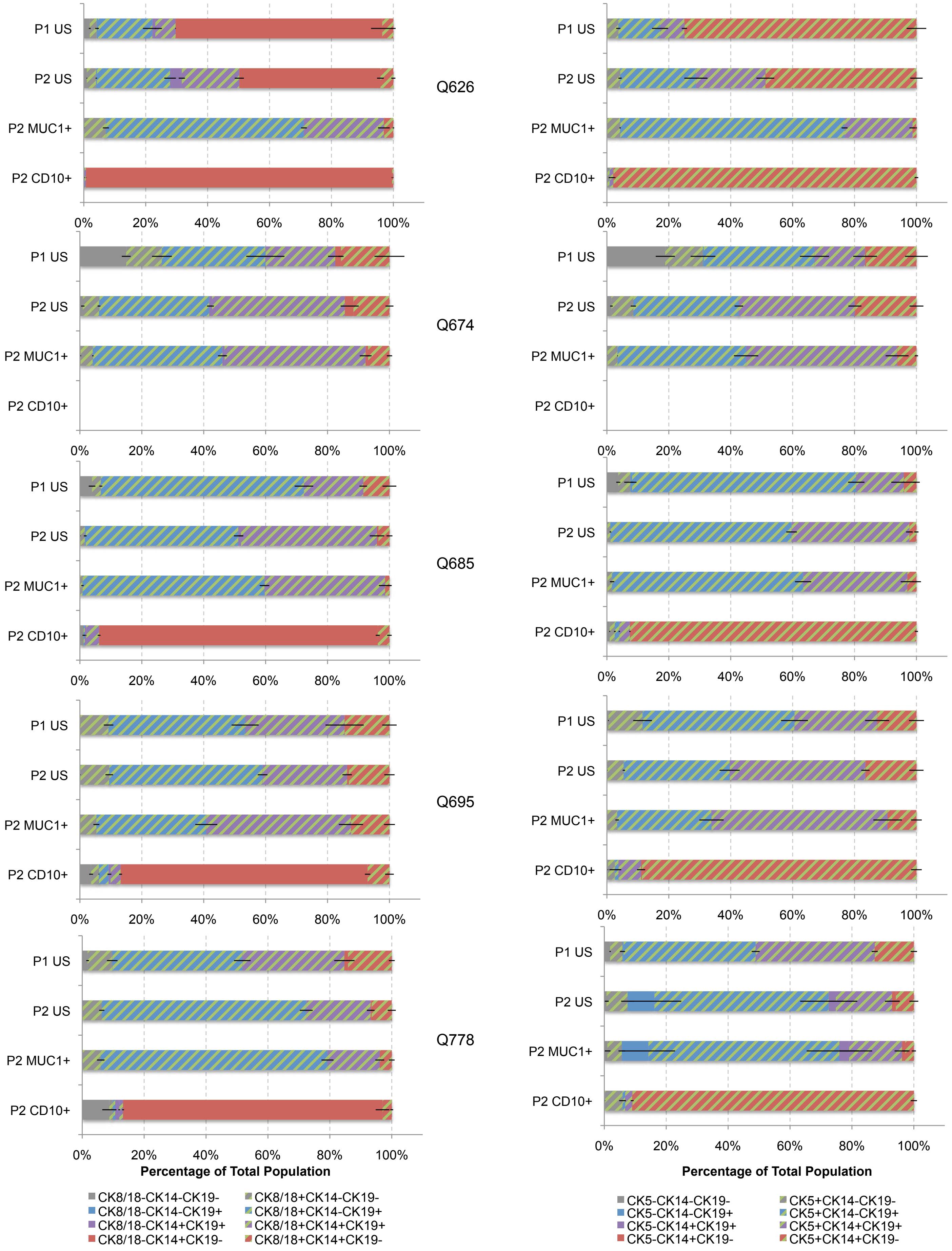

Supplementary Figure 5

A

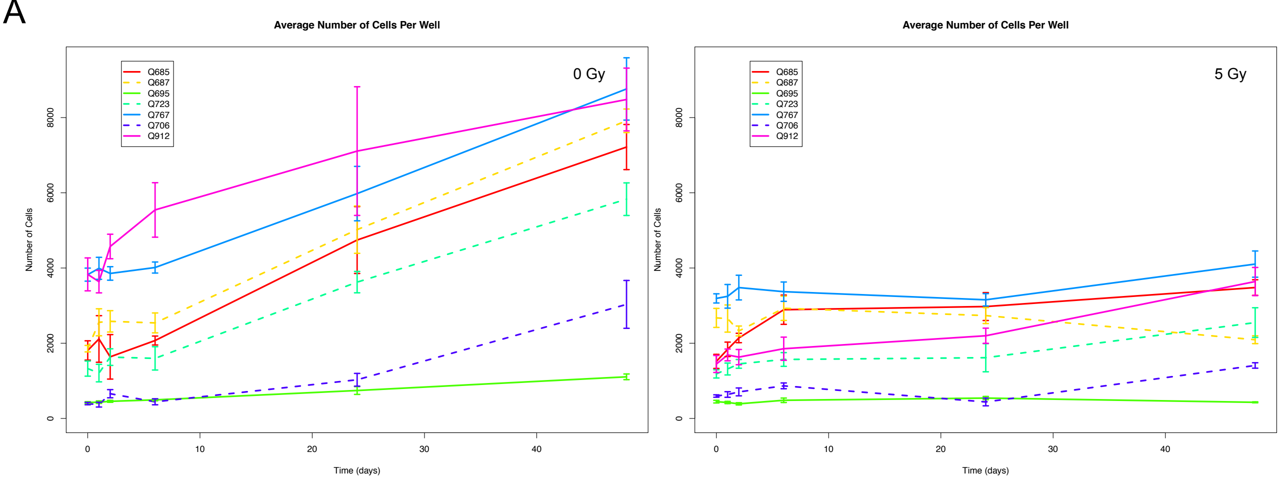

B

Split-plot analyses using linear regression (treating time as a continuous variable)

	DN		CK19+		DP		CK14+	
**Patient**	**Time**	**Txt:Time**	**Time**	**Txt:Time**	**Time**	**Txt:Time**	**Time**	**Txt:Time**
Q685	0.001	0.479	0.980	0.022	0.076	0.017	0.287	0.552
Q687	0.000	0.335	0.909	0.049	0.001	0.033	0.788	0.980
Q695	0.736	0.742	0.498	0.489	0.828	0.076	0.621	0.820
Q706	0.000	0.367	0.001	0.666	0.000	0.304	0.186	0.723
Q723	0.205	0.226	0.728	0.588	0.021	0.776	0.080	0.957
Q767	0.000	0.374	<2e-16	0.674	<2e-16	0.230	0.000	0.569
Q912	0.000	0.321	0.886	0.389	0.000	0.027	0.025	0.096

Coloured squares = comparison has achieved statistical significance

Supplementary Figure 6

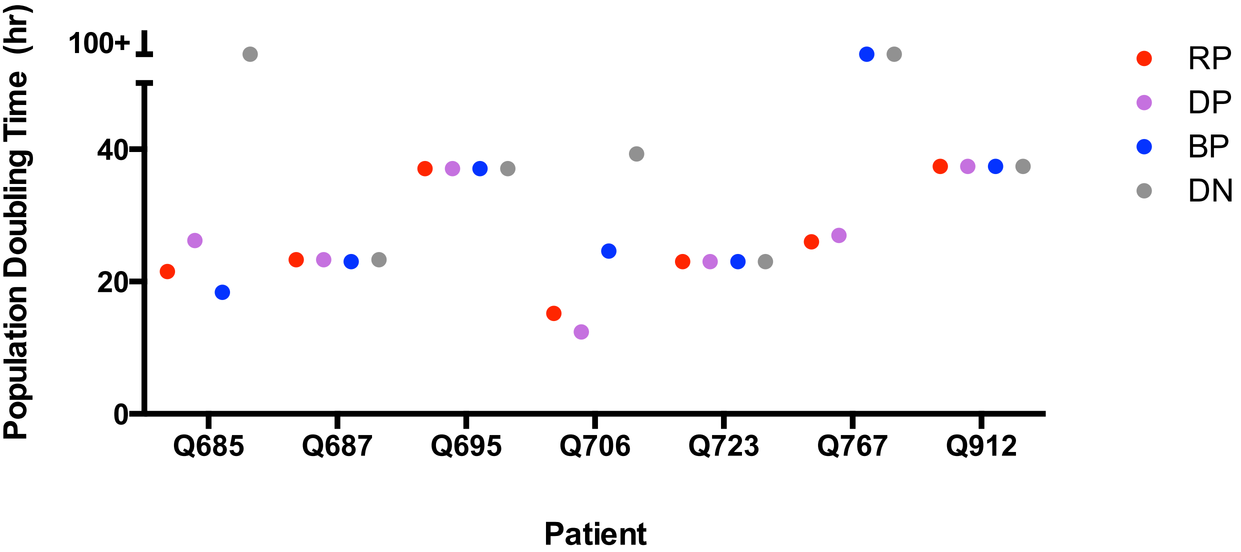

Supplementary Figure 7

A

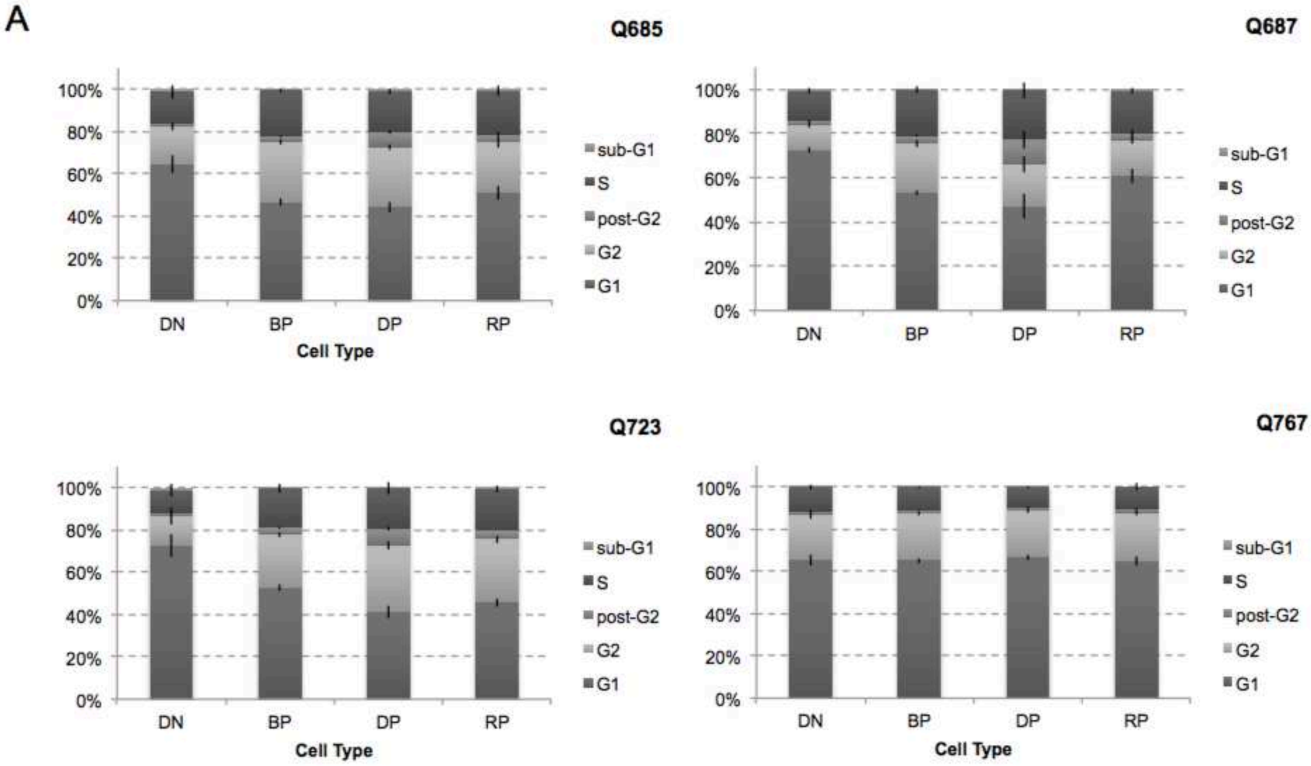

B

G1	BP	DP	RP	Patient
DN	****	****	****	Q685
	****	****	**	Q687
	****	****	****	Q723
	ns	ns	ns	Q767

ns	$p > 0.05$
*	$p \leq 0.05$
**	$p \leq 0.01$
***	$p \leq 0.001$
****	$p \leq 0.0001$

Supplementary Figure 8

		Cell type Subpopulations										Cell Cycle													yH2AX	
		Baseline cell phenotype frequency Mean (proportion)				Baseline cell phenotypes Population Doubling Time (hr)					G2/M proportions at baseline										G2M peak per cell type (hr)					
Patient ID	Age at Surgery	DN	BP	DP	RP	DN	BP	DP	RP	All	Baseline total G1	Baseline total S	Baseline total G2M	DN	BP	DP	RP	G2M peak all cells (h)	Resolves/Sustained over time	Hi DN G1 baseline	DN	BP	DP	RP	All Cells with 5 Gy treatment	Order of baseline expression
Q685	44.0	0.15	0.60	0.17	0.08	23.8	23.8	23.8	23.8	23.8	0.49	0.21	0.27	0.18	0.285	0.283	0.238	6	R	hi	24	6	24	24	S	DP, BP, RP, DN
Q687	49.5	0.14	0.74	0.09	0.03	>100	22.8	19.2	23.5	27.0	0.56	0.20	0.20	0.11	0.22	0.19	0.16	6	S	hi	6	6	6	6	S	DP, BP, RP, DN
Q695	51.0	0.13	0.61	0.10	0.15	35.4	35.4	35.4	35.4	35.4				ND				24	S	Insufficient data to stratify by cell type					R	DP, BP, RP, DN
Q706	46.9	0.16	0.16	0.17	0.51	46.1	22.2	11.2	16.7	18.5				ND							ND				R	DP, RP, BP, DN
Q723	19.2	0.06	0.48	0.20	0.26	20.4	20.4	20.4	20.4	20.4	0.50	0.19	0.27	0.14	0.251	0.313	0.298	24	R	hi	24	24	24	24	R	RP, DP, BP, DN
Q767	73.3	0.13	0.60	0.20	0.07	>100	>100	21.2	22.6	47.3	0.65	0.11	0.22	0.216	0.221	0.221	0.229	24	S	no	24	24	24	24	R	DP, BP, DN, RP
Q912	36.5	0.30	0.52	0.05	0.13	37.4	37.4	37.4	37.4	37.4				ND							ND				R	DP, RP, BP, DN
AVG		0.15	0.53	0.14	0.17	32.62	27.00	24.09	25.69	29.97	0.5	0.2	0.2	0.16	0.24	0.25	0.23									

Supplementary Figure 9

A

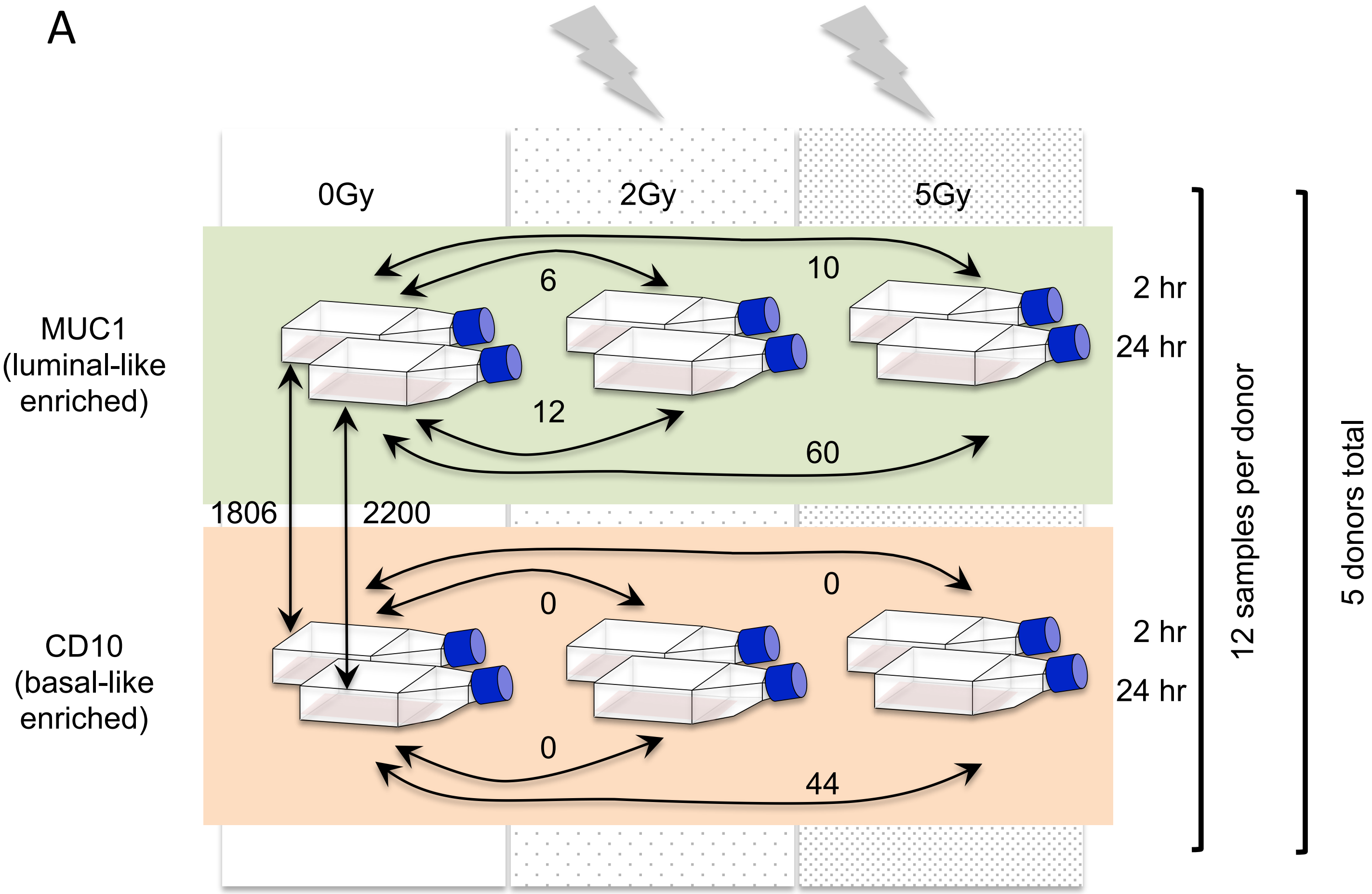

B

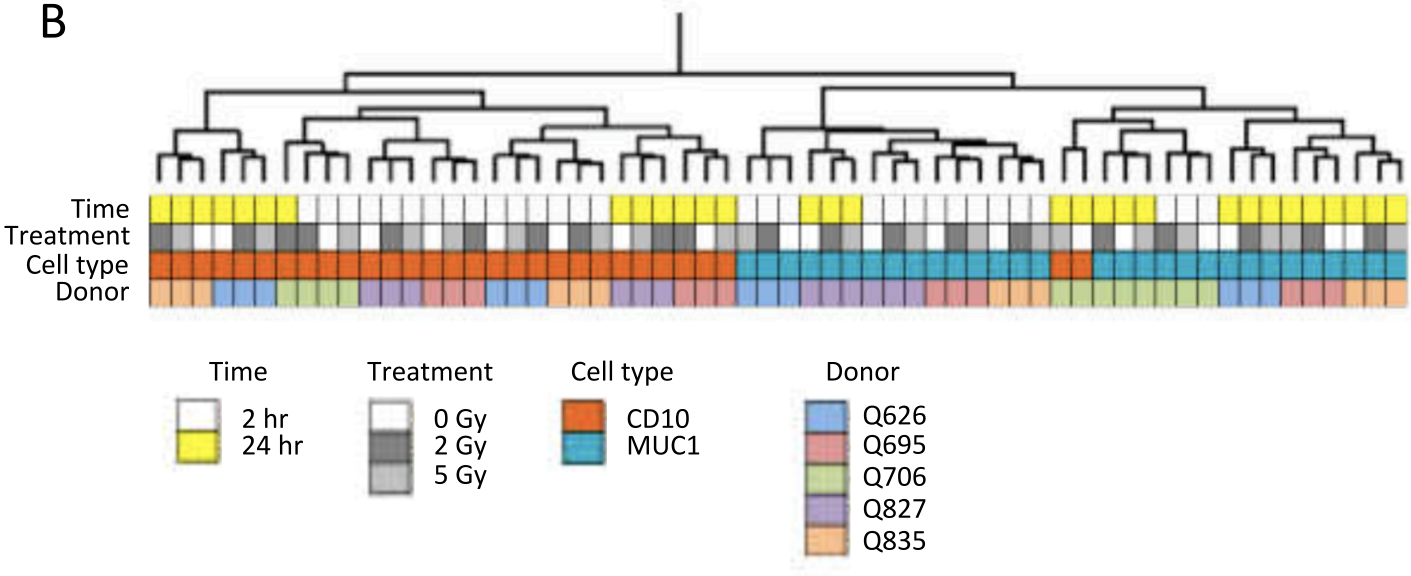

Supplementary Figure 10

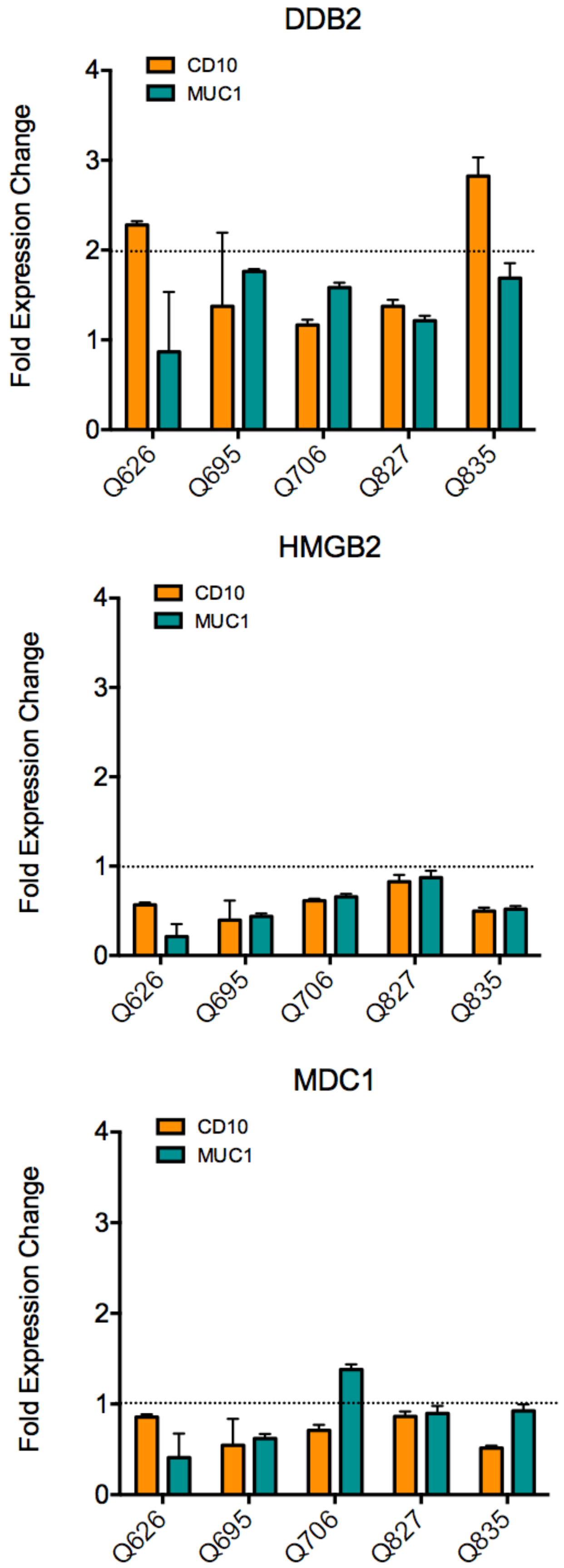

Supplementary Table 1: Ingenuity Pathway Analysis of the 5 Gy IR response in MUC+- and CD10+-sorted cells at 24 hr.

Ingenuity Pathways Analysis

	MUC1		CD10	
**Molecular and Cellular Functions**	p-value		p-value	
Cell Cycle	2.19E-37 - 1.69E-02		3.59E-17 - 1.98E-02	
Cellular Assembly and Organization	4.86E-32 - 1.55E-02		4.85E-11 - 1.98E-02	
DNA Replication, Recombination, and Repair	4.86E-32 - 1.80E-02		4.85E-11 - 1.98E-02	
Cellular Movement	1.82E-19 - 1.71E-02		7.85E-09 - 1.98E-02	
Cell Death and Survival	2.66E-10 - 1.86E-02		2.62E-08 - 1.98E-02	
**Upstream Regulators**	p-value		p-value	
TP53	9.78E-28	Act	4.92E-23	Act
NUPR1	7.12E-26	Inhib	7.38E-12	
FOXM1	9.58E-25	Inhib	2.52E-11	Inhib
E2F4	2.67E-27		7.19E-09	
RB1			6.94E-08	Inhib
FOXO1	1.88E-20	Inhib		
**Canonical Pathways**	p-value		p-value	
Mitotic Roles of Polo-Like Kinase	1.90E-15		3.31E-06	
Cell Cycle: G2/M DNA Damage Checkpoint Regulation	7.66E-09		1.87E-04	
p53 Signaling	3.03E-08		2.85E-07	
ATM Signaling	1.82E-05			
Role of CHK Proteins in Cell Cycle Checkpoint Control	9.29E-05			
Salvage Pathways of Pyrimidine Ribonucleotides			2.52E-04	
Cysteine Biosynthesis/Homocysteine Degradation			3.90E-04	

Supplementary Figure Legends

Supplementary Figure 1: Cell phenotype classification using cytokeratin intensity distributions and normal mixtures. The intensity distributions for K14 and K19 of stained populations combined with their respective negative control (black) were modeled using a mixture of normal distributions (red). The threshold for positivity (dashed red line) is made at the first intersection point of the normal distributions after the negative control distribution arrives at zero (dashed red line).

Supplementary Figure 2: Relationship between frequency of cell type in primary culture and donor age. The frequencies of DN (K14$^-$/K19$^-$), DP (K14$^+$/K19$^+$), K14$^+$ (K14$^+$/K19) and K19$^+$ (K14$^-$/K19$^+$) cell type subpopulations, as a percentage of the total population, were plotted per donor against age at time of surgery. Each data point is the mean of the technical replicates (n=5-6) and error bars are standard error. Linear regression was calculated and plotted, but no significant trends were observed within each cell type.

Supplementary Figure 3: Subpopulation frequencies defined by K14/K19 in technical replicates and independent cultures of hMECs. Quantification of CK defined subpopulations was reproducible across duplicate plates seeded in parallel (A) and even on cultures from the same donor grown independently on separate occasions (B). Similarity was assessed using 1-way ordinary ANOVA for each cell type. Bars represent the average subpopulation frequency of 5 technical replicates (wells) with standard error.

Supplementary Figure 4: Subpopulation frequencies in P1, P2, MUC1$^+$ sorted and CD10$^+$ sorted hMECs. CK5 and CK8/18 expression are shown overlaid on K14/K19 populations. Each values represents the mean frequency of the technical replicates and standard error is given.

Supplementary Figure 5: (A) The effect of 5 Gy radiation on hMEC growth over 48 hr. Data for 7 donors is shown, each data point representing the mean of the technical replicates and the error bars representing standard error. (B) ANOVA split-plot analysis testing the hypothesis that there is an interaction between time and treatment

for the nominated phenotype (Txt:Time) and the hypothesis the proportion of the nominated phenotype does not change over time regardless of treatment (Time). (C) Subpopulation proportions in donor Q687 with time and with treatment (0 Gy and 5 Gy) with linear regression. Each data point represents a technical replicate. Blue = $K19^+$; red = $K14^+$; purple = double positive; grey = double negative.

Supplementary Figure 6: Estimated populations doubling rates for each donor stratified by cell type. Each point represents the average population doubling rate calculated from 0 to 48 hr time-points.

Supplementary Figure 7: Cell cycle phase frequencies according to cell phenotype per donor (A). Stacked plots of average proportions over (n=5-6) technical replicates with standard error. (B) p-values obtained by 1-way ordinary ANOVA for comparison with the DN population are given per donor per subpopulation

Supplementary Figure 8: Summary of data for all donors for IR dose response time course assay. S = sustained; R = resolved.

Supplementary Figure 9: (A) Schematic of the experimental design and samples profiled in gene expression array experiment. Numbers indicate the number of differentially expressed genes in that particular comparison. (B) Dendrogram of sample relationship after unsupervised cluster analysis of 19472 filtered probes. Colour bars refer to sample timepoint, treatment dose, sorted cell type of origin and individual donor.

Supplementary Figure 10: Technical Validation of Gene Expression Data. Relative expression of *DDB2*, *HMGB2* and *MDC1* in MUC1 and CD10 cultures after IR treatment shown as fold change relative to untreated controls.

Supplementary Table 1 contains Ingenuity Pathway Analysis of the 5 Gy IR response in $MUC1^+$- and $CD10^+$-sorted cells at 24 hr.
